# Mathematical modelling, analysis and numerical simulation of social media addiction and depression

**DOI:** 10.1371/journal.pone.0293807

**Published:** 2024-03-12

**Authors:** Abu Safyan Ali, Shumaila Javeed, Zeshan Faiz, Dumitru Baleanu

**Affiliations:** 1 Department of Mathematics and Computer Science, University of Ferrara, Ferrara, Italy; 2 Department of Mathematics, COMSATS University Islamabad, Islamabad Campus, Islamabad, Pakistan; 3 Department of Computer Science and Mathematics, Lebanese American University, Beirut, Lebanon; 4 Department of Mathematics, Mathematics Research Center, Near East University, Nicosia, Mersin 10, Turkey; Chongqing Three Gorges University, CHINA

## Abstract

We formulate a mathematical model of social media addiction and depression (SMAD) in this study. Key aspects, such as social media addiction and depression disease-free equilibrium point (SMADDFEP), social media addiction and depression endemic equilibrium point (SMADEEP), and basic reproduction number (**R**_0_), have been analyzed qualitatively. The results indicate that if **R**_0_ < 1, the SMADDFEP is locally asymptotically stable. The global asymptotic stability of the SMADDFEP has been established using the Castillo-Chavez theorem. On the other hand, if **R**_0_ > 1, the unique endemic equilibrium point (SMADEEP) is locally asymptotically stable by Lyapunov theorem, and the model exhibits a forward bifurcation at **R**_0_ = 1 according to the Center Manifold theorem. To examine the model’s sensitivity, we calculated the normalized forward sensitivity index and conducted a Partial Rank Correlation Coefficient (PRCC) analysis to describe the influence of parameters on the SMAD. The numerical results obtained using the Fourth-order Runge-Kutta (RK-4) scheme show that increasing the number of addicted individuals leads to an increase in the number of depressed individuals.

## 1 Introduction

The documentation of information in the modern era has undergone a substantial change, largely due to the exponential growth of internet-based social media platforms. Consequently, interpersonal communication has undergone a significant transformation [1]. The psychoactive influence of networking communication has increased as a result of its increasing popularity. Extensive research has associated compulsive use of social media platforms with detrimental outcomes, including reduced efficiency, negative social interactions, and decreased life satisfaction. Information technology has significantly reshaped the nature of social communication in the past few decades, with the rapid growth of internet based social media platforms playing a pivotal role [2–5]. Social media is a crucial technology that offers individuals valuable abilities, including but not limited to knowledge accessibility, addressing issues, entrepreneurship, self-motivated education, and more when utilized effectively [6–8]. Improper utilization of social media has adverse consequences, and one of the most significant outcomes is social media addiction [9, 10]. The emotional, relational, and performance-related problems associated with social media addiction (SMA) are a cause for concern, particularly given the rising popularity of social networking sites and the limited time people spend on them [11, 12]. The misuse of social networking sites is on the rise, and this is exemplified by the alarming statistics of 68% and 73% of American adolescents using Facebook, respectively [13]. Overusing social media is linked to poor performance evaluation, difficult interpersonal connections, poor sleep, lower levels of pleasure, and hostile, anxious, and depressed moods [14–19]. Similar to addictions to drugs, liquor, and gambling, SMA may initially seem not exceptional, but It is widespread that is only getting worse and has to be addressed. The phrase “social media addiction (SMA)” is frequently used to characterize those who participate in online communication apps like Instagram, Twitter, Facebook, YouTube, and others obsessively [20, 21]. In the twenty-first century, social media addiction is a rising issue. As a result, there has been an escalation of research studies on this topic conducted in multiple countries [22, 23].

Social relationships have been impacted by the transition away from in-person encounters to virtual ones brought on by social media. Online, people can act aggressively in ways they wouldn’t in real life. Aggression and SMA have a connection on a global and national scale. Depression, abusive behavior, and mental decline can be caused by social media addiction [24]. Spending too much time on social media has been linked with depression, social distance, and FOMO (fear of missing out), all of which have been shown to have a bad influence on one’s mental health [25]. More research is required to establish the correlation between addiction and depression on social media (SMAD). The study looks at the relation between SMA, anxiety, and depressive symptoms in Turkish, as can be shown in [26]. The World Health Organization (WHO) published a paper in 2019 that addressed the possible drawbacks of excessive social media use. According to studies included in the paper, using social media excessively might cause depression, stress, and other mental health problems [27]. Many reports and studies indicate that social media addiction leads to depression [28–30]. The number of American women who fulfill the criteria for clinical depression is presently over seven million [31]. Even after considering gender variations in self-reporting behavior, a reliable study indicates that women are nearly twice as compared as men to suffer from clinical or sub clinical depression [32]. Social media addiction and depression must be treated to stop additional negative effects. Both social media addiction and depression have treatments available, including clinical depression treatment, control measures like advertisements and educational materials, and therapeutic techniques like turning off alerts, putting time restrictions in place, cutting back on internet use, and never using a smart phone before bed.

**Fig 1:**
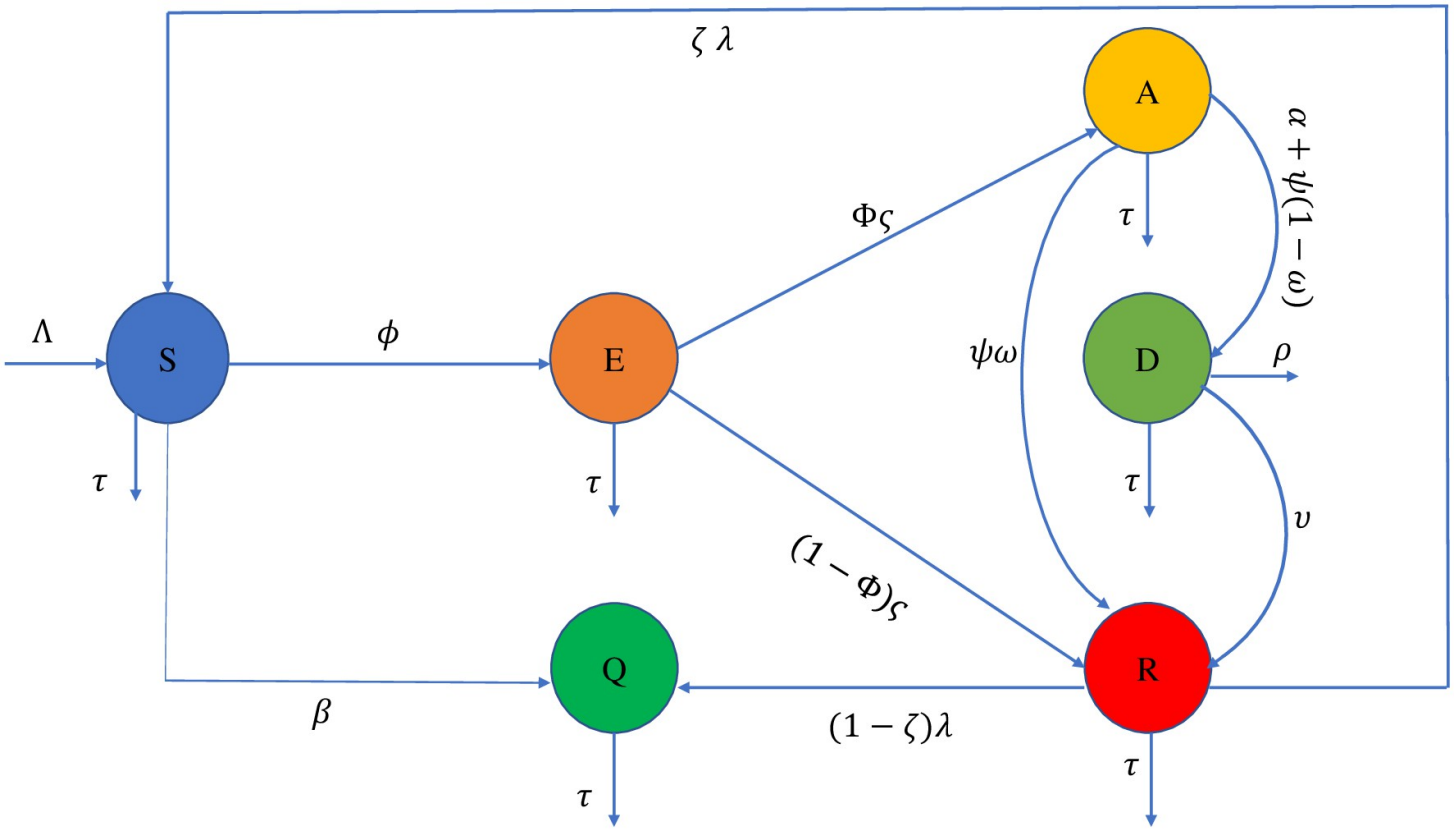
Compartmental diagram of SMAD model.

Mathematical modeling is applied to study the prevention of infectious disease dynamics and plays an important scientific contribution [33–37]. The infectious disease dynamics model has been used by many researchers to study addictions to gaming, alcohol, drugs, and social media, depression, and other issues [38–44]. Here we proposed a mathematical model of social media addiction and depression (SMAD). The purpose of this work is to examine the effects of social media addiction on the mental health of people that leads to depression of any age. The social media-addicted population can be depressed by social comparison, increasing feelings of loneliness, harassment, bad news and comments on social apps, decreasing motivation, increasing stress levels, decreasing social interaction in real, relationship problems, etc. The diagram of the SMAD model is shown in Fig 1. The paper is divided into the following sections for structure. We discuss and establish the mathematical model in section 2. The investigation of steady states, basic reproduction numbers, and strength numbers is the focus of section 3. The positive invariant region, Positivity, and Boundedness of the solution are illustrated in section 4. The stability analysis and the existence of a unique solution of the model are included in sections 5 and 6. The sensitivity analysis by Normalized forward sensitive and Partial Rank Correlation Coefficient (PRCC) techniques is the subject of section 7. On the other side, section 8 presents numerical results for both cases **R**_0_ > 1, **R**_0_ < 1. In section 9, we present a conclusion.

## 2 Social Media Addiction and Depression (SMAD) model formulation

We proposed the social media addiction depression (SMAD) mathematical model here.

$$\{\begin{array}{l} \frac{\text{d}S}{\text{d}t}=\Lambda +\zeta \lambda R-\phi \chi I_{1}S-\left(\beta +\tau \right)S,\\ \frac{\text{d}E}{\text{d}t}=\phi \chi I_{1}S-\left(\;\varsigma \;+\tau \right)E,\\ \frac{\text{d}I_{1}}{\text{d}t}=\Phi \;\varsigma \;E-\left(\tau +\psi +\alpha \right)I_{1},\\ \frac{\text{d}I_{2}}{\text{d}t}=\alpha I_{1}+\psi \left(1-\omega \right)I_{1}-\left(\upsilon +\rho +\tau \right)I_{2},\\ \frac{\text{d}R}{\text{d}t}=\left(1-\Phi \right)\;\varsigma \;E+\upsilon I_{2}+\psi \omega I_{1}-\left(\tau +\lambda \right)R,\\ \frac{\text{d}Q}{\text{d}t}=\beta S+\left(1-\zeta \right)\lambda R-\tau Q. \end{array}$$ (1)

The following are the initial conditions (ICs) at time t = 0:

$$\begin{array}{c} S\left(0\right)\geq 0,E\left(0\right)\geq 0,I_{1}\left(0\right)\geq 0,I_{2}\left(0\right)\geq 0,R\left(0\right)\geq 0,Q\left(0\right)\geq 0, \end{array}$$

Proposing a mathematical model for SMAD is based on the following hypotheses:

- The SMAD outbreak takes place in a closed atmosphere.
- The likelihood of developing a social media addiction is unaffected by gender, race, or socioeconomic standing.
- Each participant interacts with the other uniformly (to the same extent).
- Individuals who are not addicted to social media may become addicted if they are influenced by the social pressure of their peers who are addicted.
- Transmission of depression in SMA individuals is significantly impacted by the media, and this impact is either reduced or mediated by an individual difference variable like socialization or pre-existing body dissatisfaction.

In this model (1), the total individuals are categorized into six compartments: Susceptible individuals *S*: those who are not currently infected but susceptible to SMA, Exposed individuals *E*: those who use social media occasionally and are at risk of developing an addiction, Addictive individuals *I*_1_: those who used social media frequently and are addicted now, Depressed individuals *I*_2_: those who are depressed by the effect of SMA, Recovered/Removed individuals *R*: those who completed treatment and recovered from the infection, Permanent quitters individuals *Q*: those who do not use social media and/or quit social media. The total number of individuals (population) is denoted by *N* and is given by

$$\begin{array}{c} N=S+E+I_{1}+I_{2}+R+Q \end{array}$$

The recruiting of susceptible people into the population happens at a rate of Λ. These individuals are influenced by their peer pressure to use social media at a contact rate of *ϕ* from addicted individuals with the possible transmission of *χ* and join the exposed class. Some susceptibles transfer to permanent quitter at a rate of *β*. The exposed population suffers from SMA and transit to the addicted class at a rate of Φ*ς*, whereas the rest recovered by treatments at a rate (1 − Φ)*ς*. The addictive individuals become depressed at rate *α* and those who leave treatment or need a second dose at rate (1 − *ω*)*ψ* also become depressed. The depressed individuals either moved to the recovered class through education and/or treatment at a rate of *υ*, or they passed away from depression at a rate of *ρ*. The probability rate of treatment to be successful to become recovered is *ωψ*. The recovered individuals either permanently quit using social media at a rate of (1 − *ζ*)λ or they start to be susceptible again to the SMAD at a rate of *ζ* λ. The average death rate for the entire population is *τ*. The system’s parameters are displayed in the Table 1.

**Table 1:** Values of parameters involved in the proposed SMAD model (1).

Parameters Symbol	Description of parameters	Parameters values	Source
Λ	Recruitment rate of susceptible individuals	0.5	Assume
*ζ*	Percentage of people in the recovered class who are susceptible to SMAD	0.35	[45]
λ	Individuals that leave recovered class	0.4	[42]
*τ*	Natural death rate	0.05-0.25	[42, 45]
*α*	The rate at which depression is brought on by media impact	0.3-0.5	Approximate
*β*	Susceptibles who avoid and/or stop using social media	0.01	Assume
*χ*	Contact rate of susceptibles with addicted population	0.25	[42]
*υ*	Depressed individuals that move to recovered individuals through the treatment	0.7	[40]
*ς*	Individuals that leave exposed class	0.25	[40]
*ω*	Probability that treatment is successful	0.8	[43]
*ψ*	The rate at which people leave treatment	0.0027	[43]
Φ	Percentage of those who are exposed who later become addicts	0.7	[40]
*ρ*	Rate at which people suicide to depression	0.01	Assume
*ϕ*	Transmission rate of addiction to the susceptible population	0.1-0.8	[40]

## 3 Equilibrium points of SMAD model

The equilibrium points of the SMAD model are determined in this paragraph.

$$\{\begin{array}{ll} \Lambda +\zeta \lambda R-\phi \chi I_{1}S-\left(\beta +\tau \right)S & =0,\\ \phi \chi I_{1}S-\left(\;\varsigma \;+\tau \right)E & =0,\\ \Phi \;\varsigma \;E-\left(\tau +\psi +\alpha \right)I_{1} & =0,\\ \alpha I_{1}+\psi \left(1-\omega \right)I_{1}-\left(\upsilon +\rho +\tau \right)I_{2} & =0,\\ \left(1-\Phi \right)\;\varsigma \;E+\upsilon I_{2}+\psi \omega I_{1}-\left(\tau +\lambda \right)R & =0,\\ \beta S+\left(1-\zeta \right)\lambda R-\tau Q & =0, \end{array}$$ (2)

Two equilibrium points are reached in the feasible region Δ after solving the above set of Eq (2). These numbers were derived using the Maple software. SMADDFE of the model is given as

$$\begin{array}{c} E_{q0}=\left(S^{0},E^{0},{I_{1}}^{0},{I_{2}}^{0},R^{0},Q^{0}\right)=(\frac{\Lambda }{\left(\beta +\tau \right)},0,0,0,,0,\frac{\beta \;\Lambda }{(\beta +\tau )\tau }). \end{array}$$

SMADDFE will occur when *R*_0_ < 1, which means there is no infection. This indicates that only susceptible individuals and those who permanently quit will remain, while others will vanish. The SMADEEP of the system (2) is

$$\begin{array}{c} E_{q1}=(S^{1},E^{1},{I_{1}}^{1},{I_{2}}^{1},R^{1},Q^{1}). \end{array}$$

where

$$\begin{array}{c} S^{1}=\frac{\left(\tau +\;\varsigma \;)(\tau +\alpha +\psi \right)}{\;\varsigma \;\;\phi \;\chi \;\Phi }. \end{array}$$

$$\begin{array}{c} E^{1}=-((\tau +\rho +\upsilon (\tau +\alpha +\psi )(\tau +\lambda )(\tau^{3}+(\psi +\beta +\alpha +\;\varsigma \;)\tau^{2}+(\left(\psi +\beta +\alpha \right)\;\varsigma \;+\\ \beta \;\left(\psi +\alpha \right))\tau -\;\varsigma \;\;(\Lambda \;\phi \;\chi \;\Phi -\alpha \;\beta -\beta \;\psi )))/(\chi \;(\tau^{4}+\left(\lambda +\psi +\rho \alpha +\;\varsigma \;+\upsilon \right)\tau^{3}+\\ \left(\left((1+(\Phi -1)\zeta )\lambda +\psi +\rho +\alpha +\upsilon \right)\;\varsigma \;+(\psi +\rho +\alpha +\upsilon )\lambda +(\rho +\upsilon )(\psi +\alpha )\right)\tau^{2}+\\ (\left(\left(\left(1+\left(-1+\left(-\omega +1\right)\Phi \right)\zeta \right)\psi +\left(\rho +\alpha +\upsilon \right)\left(1+\left(\Phi -1\right)\zeta \right)\right)\lambda +\left(\rho +\upsilon \right)(\psi +\alpha )\right)\;\varsigma \;+\\ \lambda \;(\rho +\upsilon )(\psi +\alpha ))\tau -\lambda \;(\left((-1+(1+(\omega -1)\Phi )\zeta )\rho +\upsilon \;(\zeta -1)\right)\psi -\alpha \;((1+(\Phi -1)\zeta \\ \rho -\upsilon \;(\zeta -1)))\;\varsigma \;)\phi \;\Phi \;\;\varsigma \;). \end{array}$$

$$\begin{array}{c} {I_{1}}^{1}=-\left((\tau +\rho +\upsilon )(\tau +\lambda )(\tau^{3}+(\psi +\beta +\alpha +\;\varsigma \;)\tau^{2}+((\psi +\beta +\alpha \right)\;\varsigma \;\\ +\beta \;(\psi +\alpha ))\tau -\;\varsigma \;\;(\Lambda \;\phi \;\chi \;\Phi -\alpha \;\beta -\beta \;\psi )))/(\phi \;(\tau^{4}+(\lambda +\psi +\rho +\alpha +\;\varsigma \;+\upsilon )\tau^{3}\\ +(((1+(\Phi -1)\zeta )\lambda +\psi +\rho +\alpha +\upsilon )\;\varsigma \;+(\psi +\rho +\alpha +\upsilon )\lambda +(\rho +\upsilon )(\psi +\alpha ))\tau^{2}\\ +\left((((1+(-1+(-\omega +1)\Phi )\zeta )\psi +(\rho +\alpha +\upsilon \right)\left(1+\Phi \;\zeta -\zeta \right))\lambda +(\rho +\upsilon )(\psi +\alpha ))\\ \;\varsigma \;+\lambda \;(\rho +\upsilon )(\psi +\alpha ))\tau -\lambda \;(\left(\left(-1+\left(1+\left(\omega -1\right)\Phi \right)\zeta \right)\rho +\upsilon \;\left(\zeta -1\right)\right)\\ \psi -\alpha \;\left(\left(1+\left(\Phi -1\right)\zeta \right)\rho -\upsilon \;\left(\zeta -1\right)\right))\;\varsigma \;)\chi ). \end{array}$$

$$\begin{array}{c} {I_{2}}^{1}=(((\omega -1)\psi -\alpha )(\tau +\lambda )(\tau^{3}+(\psi +\beta +\alpha +\;\varsigma \;)\tau^{2}+((\psi +\beta +\alpha )\;\varsigma \\ +\beta \;(\psi +\alpha ))\tau -\;\varsigma \;\;(\Lambda \;\phi \;\chi \;\Phi -\alpha \;\beta -\beta \;\psi )))/(\phi \;(\tau^{4}+(\lambda +\psi +\rho +\alpha +\;\varsigma \;+\upsilon )\tau^{3}+\\ (((1+(\Phi -1)\zeta )\lambda +\psi +\rho +\alpha +\upsilon )\;\varsigma \;+(\psi +\rho +\alpha +\upsilon )\lambda +(\rho +\upsilon )(\psi +\alpha ))\tau^{2}+\\ ((((1+(-1+(-\omega +1)\Phi )\zeta )\psi +(\rho +\alpha +\upsilon )(1+(\Phi -1)\zeta ))\lambda +(\rho +\upsilon )(\psi +\alpha ))\;\varsigma \;+\lambda \\ (\rho +\upsilon )(\psi +\alpha ))\tau -\lambda \;\left(\left((-1+(1+(\omega -1)\Phi )\zeta )\rho +\upsilon \;\left(\zeta -1\right)\right)\psi -\alpha \;((1+(\Phi -1)\zeta \right)\\ \rho -\upsilon \;(\zeta -1)))\;\varsigma \;)\chi ). \end{array}$$

$$\begin{array}{c} R^{1}=((\tau^{3}+\left(\psi +\beta +\alpha +\;\varsigma \;\right)\tau^{2}+\left((\psi +\beta +\alpha )\;\varsigma \;+\beta \;(\psi +\alpha )\right)\tau -\;\varsigma \\ \;(\Lambda \;\phi \;\chi \;\Phi -\alpha \;\beta -\beta \;\psi ))(\left(\Phi -1\right)\tau^{2}+\left((-1+(-\omega +1)\Phi )\psi +(\rho +\alpha +\upsilon )(\Phi -1)\right)\tau +\\ \left((-1+(-\omega +1)\Phi )\rho -\upsilon \right)\psi +\alpha \;\left(\left(\Phi -1\right)\rho -\upsilon \right)))/(\chi \;(\tau^{4}+\left(\lambda +\psi +\rho +\alpha +\;\varsigma \;+\upsilon \right)\tau^{3}\\ +\left(\left((\zeta \;\Phi -\zeta +1)\lambda +\psi +\rho +\alpha +\upsilon \right)\;\varsigma \;+\left(\psi +\rho +\alpha +\upsilon \right)\lambda +(\rho +\upsilon )(\psi +\alpha )\right)\tau^{2}+\\ (\left(\left(\left(-\zeta \;(\omega -1)\Phi -\zeta +1\right)\psi +(\rho +\alpha +\upsilon )\left(\zeta \;\Phi -\zeta +1\right)\right)\lambda +\left(\rho +\upsilon \right)(\psi +\alpha )\right)\;\varsigma \;+\\ \lambda \;(\rho +\upsilon )(\psi +\alpha ))\tau -(\left((\zeta \;(\omega -1)\Phi +\zeta -1)\rho +\upsilon \;(\zeta -1)\right)\psi -((\zeta \;\Phi -\zeta +1)\rho -\upsilon \\ \left(\zeta -1)\right)\alpha )\;\varsigma \;\;\lambda )\phi \;\Phi ). \end{array}$$

$$\begin{array}{c} Q^{1}=(\beta \;\tau^{6}+((-(\Phi -1)(\zeta -1)\lambda +2\;\beta )\;\varsigma \;+\beta \;(\lambda +2\;\psi +\rho +2\;\alpha +\upsilon ))\tau^{5}+\\ (\left(-(\Phi -1)(\zeta -1)\lambda +\beta \right){\;\varsigma \;}^{2}+((\left(2+(\omega -2)\Phi \right)(\zeta -1)\psi +((-2\;\zeta +2)\alpha +\\ \left(-\zeta +1)\rho -\zeta \;\upsilon +\beta +\upsilon \right)\Phi +\left(2\;\zeta -2\right)\alpha +(\zeta -1)\rho +\zeta \;\upsilon +\beta -\upsilon )\lambda +4\\ \left(\psi +\rho /2+\alpha +\upsilon /2)\beta \right)\;\varsigma \;+2\;\left(\left(\psi +\rho /2+\alpha +\upsilon /2\right)\lambda +1/2\;(\psi +\alpha )(\psi +2\;\rho +\alpha +2\;\upsilon )\right)\beta )\tau^{4}+\\ ((\left((2+(\omega -2)\Phi )(\zeta -1)\psi +\left((-2\;\zeta +2)\alpha +(-\zeta +1)\rho -\zeta \;\upsilon +\beta +\upsilon \right)\Phi +(\rho +2\;\alpha +\upsilon )(\zeta -1)\right)\\ \lambda +2\;(\psi +\rho /2+\alpha +\upsilon /2)\beta ){\;\varsigma \;}^{2}+(((\zeta -1)(1+(\omega -1)\Phi )\psi^{2}+(((\omega -2)(\zeta -1)\alpha +(\omega -2)(\zeta -1)\rho +\\ \left(-\omega +2)\beta -\upsilon \;(\zeta -1)\right)\Phi +(2\;\zeta -2)\alpha +(2\;\zeta -2)\rho +2\;\zeta \;\upsilon +2\;\beta -2\;\upsilon )\psi +\left((-\zeta +1)\alpha^{2}+((-2\;\zeta +2\right)\\ \rho -\zeta \;\upsilon +2\;\beta +\upsilon )\alpha +\beta \;(\rho +\upsilon ))\Phi +(\zeta -1)\alpha^{2}+\left((2\;\zeta -2)\rho +2\;\zeta \;\upsilon +2\;\beta -2\;\upsilon \right)\alpha +\beta (\rho +\upsilon ))\lambda +2\;\beta \\ \left(\psi +\alpha )(\psi +2\;\rho +\alpha +2\;\upsilon ))\;\varsigma \;+(\psi +\alpha )\beta \;\left((\psi +2\;\rho +\alpha +2\;\upsilon )\lambda +(\rho +\upsilon )(\psi +\alpha )\right)\right)\tau^{3}+\\ ((((\zeta -1)(1+(\omega -1)\Phi )\psi^{2}+(\left((\omega -2)(\zeta -1)\alpha +(\omega -2)(\zeta -1)\rho +\left(-\omega +2\right)\beta -\upsilon \;(\zeta -1)\right)\Phi +2\\ \left(\rho +\alpha +\upsilon )(\zeta -1)\right)\psi +\chi \;\Lambda \;\phi \;(\zeta -1)\Phi^{2}+((-\zeta +1)\alpha^{2}+\left(\left(-2\;\zeta +2\right)\rho -\zeta \;\upsilon +2\;\beta +\upsilon \right)\alpha +\rho \;\beta +\beta \\ \upsilon -\chi \;\Lambda \;\phi \;(\zeta -1))\Phi +2\;\alpha \;(\rho +\alpha /2+\upsilon )(\zeta -1))\lambda +\beta \;(\psi +\alpha )\left(\psi +2\;\rho +\alpha +2\;\upsilon \right)){\;\varsigma \;}^{2}+\\ ((\left(((\zeta -1)\rho -\beta )(\omega -1)\Phi +(\zeta -1)\rho +\zeta \;\upsilon +\beta -\upsilon \right)\psi^{2}+(((\omega -2)\left(\left(\zeta -1\right)\rho -\beta \right)\alpha -\\ \left((\omega -2)\rho -\upsilon \right)\beta )\Phi +((2\;\zeta -2)\rho +2\;\zeta \;\upsilon +2\;\beta -2\;\upsilon )\alpha +2\;\beta \;(\rho +\upsilon ))\psi -((\left((\zeta -1)\rho -\beta \right)\\ \alpha -2\;(\rho +\upsilon /2)\beta )\Phi +((-\zeta +1)\rho -\zeta \;\upsilon -\beta +\upsilon )\alpha -2\beta (\rho +\upsilon ))\alpha )\lambda +2\;\beta \;{\left(\psi +\alpha \right)}^{2}(\rho +\upsilon ))\;\varsigma \;+\lambda \;\beta \\ {\left(\psi +\alpha \right)}^{2}(\rho +\upsilon ))\tau^{2}-(((\left(-((\zeta -1)\rho -\beta )(\omega -1)\Phi -(\rho +\upsilon )(\zeta -1)\right)\psi^{2}+(\chi \;\Lambda \;\phi \;(\omega -1)(\zeta -1)\Phi^{2}+\\ \left(-\left(\omega -2\right)((\zeta -1)\rho -\beta )\alpha +\beta \;(\omega -2)\rho -\beta \;\upsilon +\chi \;\Lambda \;\phi \;(\zeta -1)\right)\Phi -2\;\alpha \;(\rho +\upsilon )(\zeta -1))\psi -\chi \;\Lambda \;\phi \\ (\rho +\alpha +\upsilon )(\zeta -1)\Phi^{2}+\left(((\zeta -1)\rho -\beta )\alpha^{2}+\left(-2\;\rho \;\beta -\beta \;\upsilon +\chi \;\Lambda \;\phi \;(\zeta -1)\right)\alpha +\chi \;\Lambda \;\phi \;(\rho +\upsilon )(\zeta -1)\right)\\ \Phi -\alpha^{2}(\rho +\upsilon )(\zeta -1))\lambda -\beta \;{\left(\psi +\alpha \right)}^{2}(\rho +\upsilon ))\;\varsigma \;+\lambda \;(\psi +\alpha )\left(\left(\rho \;(\omega -1)\Phi -\rho -\upsilon \right)\psi -\alpha \;(\rho \;\Phi +\upsilon +\rho )\right)\beta )\\ \varsigma \;\;\tau -(\rho \;\beta \;(\omega -1)\psi^{2}+\left(\chi \;\Lambda \;\phi \;\rho \;(\omega -1)(\zeta -1)\Phi +\beta \;(\omega -2)\rho \;\alpha +\chi \;\Lambda \;\phi \;\left(\rho +\upsilon \right)(\zeta -1)\right)\psi -(\chi \;\Lambda \;\phi \;\rho \;(\zeta -1)\Phi +\\ \rho \;\beta \;\alpha -\chi \;\Lambda \;\phi \;(\rho +\upsilon )(\zeta -1))\alpha )\lambda \;\Phi \;{\;\varsigma \;}^{2})/(\chi \;(\tau^{4}+\left(\lambda +\psi +\rho +\alpha +\;\varsigma \;+\upsilon \right)\tau^{3}+((\left(\zeta \;\Phi -\zeta +1\right)\\ \lambda +\psi +\rho +\alpha +\upsilon )\;\varsigma \;+\left(\psi +\rho +\alpha +\upsilon \right)\lambda +(\rho +\upsilon )(\psi +\alpha ))\tau^{2}+(((\left(-\zeta \;(\omega -1)\Phi -\zeta +1\right)\psi +\\ \left(\rho +\alpha +\upsilon )(\zeta \;\Phi -\zeta +1)\right)\lambda +(\rho +\upsilon )(\psi +\alpha ))\;\varsigma \;+\lambda \;(\rho +\upsilon )(\psi +\alpha ))\tau -\lambda \;((\zeta \;\rho \;(\omega -1)\Phi +\\ \left(\rho +\upsilon )(\zeta -1)\right)\psi -\alpha \;(\zeta \;\Phi \;\rho -(\rho +\upsilon )(\zeta -1)))\;\varsigma \;)\tau \;\phi \;\Phi \;\;\varsigma \;). \end{array}$$

### 3.1 Threshold parameter R_0_ of SMAD model

The disease’s propagation is measured using basic reproduction number **R**_0_. **R**_0_ refers to the infected population that can be generated by an infected individual during their infectious period. **R**_0_ of the model plays a vital role in analyzing the stability of steady states. If **R**_0_ < 1, there won’t be an epidemic in the population, and if **R**_0_ > 1, the epidemic will continue in the population. The next generation matrix (NGM) technique is applied to calculate the **R**_0_ and according to the approach, system (1) can be expressed as:

$$\{\begin{array}{l} \frac{\text{d}E}{\text{d}t}=\phi \chi SI_{1}-\;\varsigma \;E-\tau E,\\ \frac{\text{d}I_{1}}{\text{d}t}=\Phi \;\varsigma \;E-\tau I_{1}-\psi I_{1}-\alpha I_{1},\\ \frac{\text{d}I_{2}}{\text{d}t}=\alpha I_{1}+\psi \left(1-\omega \right)I_{1}-\left(\upsilon +\rho +\tau \right)I_{2},\\ \frac{\text{d}R}{\text{d}t}=\left(1-\Phi \right)\;\varsigma \;E+\upsilon I_{2}+\psi \omega I_{1}-\left(\tau +\lambda \right)R. \end{array}$$ (3)

Then, We get

$$F=\left(\begin{array}{c} \phi \chi SI_{1}\\ 0\\ 0\\ 0 \end{array}\right)\;\;,\;\;\;\;\;V=\left(\begin{array}{c} \left(\;\varsigma \;+\tau \right)E\\ -\Phi \;\varsigma \;E+\left(\tau +\psi +\alpha \right)I_{1}\\ -\alpha I_{1}-\psi \left(1-\omega \right)I_{1}+\left(\upsilon +\rho +\tau \right)I_{2}\\ -\left(1-\Phi \right)\;\varsigma \;E-\upsilon I_{2}-\psi \omega I_{1}+\left(\tau +\lambda \right)R) \end{array}\right)$$

The Jacobi matrices of **F** and **V** at SMADDFE, represented by F and V are provided below:

$$\begin{array}{c} F=\left(\begin{array}{cccc} 0 & \frac{\Lambda \phi \chi }{\beta +\tau } & 0 & 0\\ 0 & 0 & 0 & 0\\ 0 & 0 & 0 & 0\\ 0 & 0 & 0 & 0 \end{array}\right), \end{array}V=\left(\begin{array}{cccc} \;\varsigma \;+\tau & 0 & 0 & 0\\ -\;\varsigma \;\;\Phi & \tau +\psi +\alpha & 0 & 0\\ 0 & -\alpha -\psi \;\left(1-\omega \right) & \upsilon +\rho +\tau & 0\\ -\left(1-\Phi \right)\;\varsigma \; & -\psi \;\omega & -\upsilon & \tau +\lambda \end{array}\right)$$

The product matrix $F\;V^{-1}$ contains the largest eigenvalue, which is the model’s next generation matrix and is shown by

$$\begin{array}{c} R_{0}=\frac{\Lambda \;\;\varsigma \;\;\phi \;\chi \;\Phi }{\left(\alpha +\psi +\tau )\;(\beta +\tau )\;(\;\varsigma \;+\tau \right)}. \end{array}$$

### 3.2 Strength number of SMAD model

Reproduction has been an important idea in epidemiological modeling during the past few decades since it is a helpful computation for evaluating reproduction in certain infectious diseases. As it may be determined by calculating the nonlinear component of infected classes. As the theory suggested, one will find two component **F** and **V**, then (**FV**^−1^ − λ*I*) = 0 will be used to reproduce the reproductive number. The **F** factor is very interesting because it is obtained from the non-linear terms of the infected class of the model.

$$\begin{array}{c} \frac{\partial }{\partial I}(\frac{I}{N})=\frac{\left[N-I\right]}{N^{2}}\\ \frac{\partial^{2}}{\partial I^{2}}(\frac{I}{N})=-2\;\frac{\left[N-I\right]}{N^{3}} \end{array}$$

Considering SMADDFEP, We get

$$\begin{array}{c} =\frac{-2}{{\left(\frac{\Lambda (\tau +\beta )}{(\beta +\tau )\tau }\right)}^{2}} \end{array}$$

$$\begin{array}{c} F=(\begin{array}{c} \frac{-2\;\tau^{2}\;\phi \;\chi }{\Lambda (\tau +\beta )}\\ 0\\ 0\\ 0 \end{array}) \end{array}$$

Then

$$\begin{array}{c} det(FV^{-1}-\lambda I)=0 \end{array}$$

Resulting in

$$\begin{array}{c} A<0. \end{array}$$

Strength Number **A** is always negative indicating that the disease spread will not exhibit any renewal process and will have a single magnitude, leading to its eventual extinction.

## 4 Positively invariant region, positivity and boundedness of solution of SMAD model

### 4.1 Positively invariant region

From biological considerations, we study model (1) in the closed set

$$\begin{array}{c} \Delta =\{(S,E,I_{1},I_{2},R,Q)\in {ℜ}_{+}^{6}:0<N\leq \frac{\Lambda }{\tau }\}. \end{array}$$

where ${ℜ}_{+}^{6}$ shows the lower-dimensional faces of the non-negative cone. Δ is evidently positively invariant with respect to model (1) [40, 42].

**Theorem**

The Δ is positively invariant for model (1).

**Proof**

Assume that

$$\begin{array}{c} N=S+E+I_{1}+I_{2}+R+Q \end{array}$$

Then

$$\begin{array}{c} N^{\prime }=S^{\prime }+E^{\prime }+I_{1}^{\prime }+I_{2}^{\prime }+R^{\prime }+Q^{\prime } \end{array}$$

So, consider the model (1):

$$\begin{array}{c} \frac{\text{d}N}{\text{d}t}\leq \Lambda -\tau \;N-\rho \;I_{2} \end{array}$$ (4)

which yields that

$$\begin{array}{c} \frac{\text{d}N}{\text{d}t}\leq \Lambda -\tau \;N \end{array}$$ (5)

Hence, it suggests that $\frac{\text{d}N}{\text{d}t}<0$ as often as $N>\frac{\Lambda }{\tau }$. Thus, we have $\frac{\text{d}N}{\text{d}t}$ is bounded by Λ − *τ*.

Using the method of integrating factor on the inequality (5), We get

$$\begin{array}{c} \frac{\text{d}}{\text{d}t}\left(e^{\tau \;t}N\right)\leq e^{\tau \;t}\;\Lambda \end{array}$$ (6)

With IC’s, Integrating the Eq (6), We get

$$\begin{array}{c} N\left(t\right)\leq N\left(0\right)\;e^{-\tau \;t}+\frac{\Lambda }{\tau }\left(1-e^{-\tau \;t}\right) \end{array}$$

Suppose *t* → ∞, we obtain

$$\begin{array}{c} N\leq \frac{\Lambda }{\tau } \end{array}$$

As a result, Δ is positively invariant of the model (1), meaning that none of its boundaries are crossed by any solution path. This demonstrates that the developed model is applicable from a mathematical and epidemiological perspective. The model is regarded as being within the range of biological feasibility. It is necessary to demonstrate that every phase trajectory that began anywhere in the feasible region Δ of the phase space ultimately enters Δ and stays there. It is possible to achieve this by demonstrating the system’s global attraction and positively invariant set.

### 4.2 Positivity of SMAD model’s solutions

Here, we provide the non-negativity of the model’s solution (1) by calculating the positivity of the solution. It is important to establish that all of the state variables in the equations of the model (1) are non-negative ∀ *t* for it to have epidemiological significance [40–42]. To prove the positivity of the SMAD model’s solution, we considered the following theorem.

**Theorem**

As considering the ICs presented in (1), the solution of SMAD model (1) is positive for all *t* > 0.

**Proof**

By using the first Eq in the system of Eq (1), it is assumed that

$$\begin{array}{c} \frac{\text{d}S}{\text{d}t}=\Lambda +\zeta \;\lambda \;R-\phi \;\chi \;I_{1}S-(\beta +\tau )S\leq \Lambda -\left(\beta +\tau \right)S \end{array}$$

$$\begin{array}{c} \frac{\text{d}S}{\text{d}t}\leq \Lambda -\left(\beta +\tau \right)S \end{array}$$

Thus, We have

$$\begin{array}{c} \frac{\text{d}S}{\text{d}t}+\left(\beta +\tau \right)S\leq \Lambda \end{array}$$ (7)

By taking I.F = *e*^(*β*+*τ*)*t*^. Multiplying on both sides of Eq (7) and integrate, We get

$$\begin{array}{c} e^{(\beta +\tau )\;t}\;\frac{\text{d}S}{\text{d}t}+\left(\beta +\tau \right)S\;e^{(\beta +\tau )\;t}\leq \Lambda \;e^{(\beta +\tau )\;t} \end{array}$$

$$\begin{array}{c} \int \frac{d}{dt}(S\;e^{(\beta +\tau )t})\leq \int \Lambda \;e^{(\beta +\tau )\;t} \end{array}$$

$$\begin{array}{c} S\;e^{(\beta +\tau )t}\leq \frac{\Lambda \;e^{(\beta +\tau )\;t}}{\beta +\tau }+\;k \end{array}$$ (8)

At t = 0, we have

$$\begin{array}{c} S(0)\;\leq \frac{\Lambda \;}{\beta +\tau }+\;k \end{array}$$

By using Eq (8), we get

$$\begin{array}{c} S\;e^{(\beta +\tau )t}\leq S(0)\;+\frac{\Lambda \;}{\beta +\tau }(e^{(\beta +\tau )t}-1) \end{array}$$

$$\begin{array}{c} S\leq S(0)e^{-(\beta +\tau )t}\;+\frac{\Lambda \;}{\beta +\tau }(1-e^{-(\beta +\tau )t}) \end{array}$$ (9)

When *t* → ∞, we get

$$\begin{array}{c} \frac{\Lambda \;}{\beta +\tau }⩾S(t)⩾0 \end{array}$$ (10)

By the same procedure, we can show that

$$\begin{array}{c} E\geq 0,I_{1}\geq 0,I_{2}\geq 0,R\geq 0,Q\geq 0 \end{array}$$

This indicates that the solution of model (1) is positive.

### 4.3 Boundedness of SMAD model’s solutions

The Boundedness of the solution is covered in this section. The obtained solutions remain bounded in a feasible region Δ [42, 46].

**Theorem**

All the solutions of the model (1) are bounded.

**Proof**

The total population is denoted by *N* and can be defined as

$$\begin{array}{c} N=S+E+I_{1}+I_{2}+R+Q. \end{array}$$ (11)

Differentiating w.r.t *t*, we get

$$\begin{array}{c} N^{\prime }=S^{\prime }+E^{\prime }+I_{1}^{\prime }+I_{2}^{\prime }+R^{\prime }+Q^{\prime } \end{array}$$

By combining all of the model system’s equations (2), we get

$$\begin{array}{c} \frac{\text{d}N}{\text{d}t}=\Lambda -\tau \;N-\rho \;I_{2}. \end{array}$$ (12)

Suppose, for any initial condition $N\left(0\right)\leq \frac{\Delta }{\tau }$, where *N*(0) = *S*(0) + *E*(0) + *I*_1_(0) + *I*_2_(0) + *R*(0) + *Q*(0), we get

$$\begin{array}{c} N\leq \frac{\Lambda }{\tau }\;\;\;\;\;\;\;\;for\;all\;\;\;t\geq 0. \end{array}$$

From Eq (12), we have

$$\begin{array}{c} \frac{\text{d}N}{\text{d}t}\leq \Lambda -\tau \;N. \end{array}$$

Using Gronwall’s inequality, we get

$$\begin{array}{c} N\leq \frac{\Delta }{\tau }+(N\left(0\right)-\frac{\Delta }{\tau })e^{-\tau \;t}. \end{array}$$

Hence $N\leq \frac{\Delta }{\tau }$, for all *t* ≥ 0, whenever $N\left(0\right)\leq \frac{\Delta }{\tau }$.

Clearly,

$$\begin{array}{c} \underset{t\to \infty }{\text{lim}}\;\text{inf}\;N\leq \underset{t\to \infty }{\text{lim}}\;\text{sup}\;N\leq \frac{\Delta }{\tau }>0. \end{array}$$

This indicates that the solution of model (1) is bounded in the feasible region Δ. This shows that the feasible area Δ contains the positive solution to the model (1).

## 5 Uniqueness and existence of SMAD model’s solution

The general 1st order ODE has the following form:

$$\begin{array}{c} \frac{\text{d}y}{\text{d}t}=g\left(y,t\right)\;\;\;\;\;\;\;\;x\left(t_{0}\right)=y_{0} \end{array}$$ (13)

The following inquiries will be of interest to one:

- What conditions allow us to declare that the solution of Eq (13) exists?
- Under what conditions allow us to declare that there is a unique solution to Eq (13)?

For this, let

$$\{\begin{array}{l} g_{1}=\Lambda +\zeta \lambda R-\phi \chi SI_{1}-\left(\beta +\tau \right)S,\\ g_{2}=\phi \chi I_{1}S-\;\varsigma \;E-\tau E,\\ g_{3}=\Phi \;\varsigma \;E-\tau I_{1}-\psi I_{1}-\alpha I_{1},\\ g_{4}=\alpha I_{1}+\psi \left(1-\omega \right)I_{1}-\left(\upsilon +\rho +\tau \right)I_{2},\\ g_{5}=\left(1-\Phi \right)\;\varsigma \;E+\upsilon I_{2}+\psi \omega I_{1}-\left(\tau +\lambda \right)R,\\ g_{6}=\beta S+\left(1-\zeta \right)\lambda R-\tau Q. \end{array}$$

The existence and uniqueness of our SMAD model’s solution are shown using the given theorem.

**Theorem(Uniqueness of SMAD Model’s Solution)**

Let’s Ω denote the domain:

$$\begin{array}{c} \Omega =(|t-t_{0}|\leq a,|x-x_{0}|\leq b,y=\left(y_{1},y_{2},y_{3},\ldots ‥y_{n}\right),y_{0}=\left(y_{10},y_{20},y_{30},\ldots ‥y_{n0}\right)) \end{array}$$ (14)

and assume that g(t,y) satisfies the Lipschitz condition:

$$\begin{array}{c} ||g\left(t,y_{1}\right)-g\left(t,y_{2}\right)\left|\right|\leq L||y_{1}-y_{2}\left|\right| \end{array}$$ (15)

and whenever (*t*, *y*_1_), (*t*, *y*_2_) ∈ Ω, where k represent any positive constant value. Then, ∃ a *δ* > 0 s.t ∃ a unique continuous vector solution y(t) of the system (13) in the interval |*t* − *t*_0_| ≤ *δ*. It is important to remember that Eq (15) is achieved by the condition that:

$$\{\frac{\partial g_{i}}{\partial y_{j}},\;\;\;\;\;i,j=1,2,3,\ldots .n$$ (16)

be continuous and bounded in the domain Ω [47].

**Lemma**

If partial derivative of *g*(*t*, *y*) i.e $\frac{\partial g_{i}}{\partial y_{j}}$ is continuous on a bounded closed convex set of real numbers domain ℜ, then it satisfies a Lipschitz condition in ℜ, where ℜ represent real numbers. Domain of our interest is 1 ≤ *ϵ* ≤ ℜ, 0 < ℜ < ∞.

**Theorem(Existence of SMAD Model’s Solution)**

Let Ω represent the domain defined in (14) s.t (15) and 1 ≤ *ϵ* ≤ ℜ hold. Then ∃ a solution of (1) which is bounded in the domain Ω [47].

**Proof**

Suppose

$$\begin{array}{c} g_{1}=\Lambda +\zeta \;\lambda \;R-\phi \;\chi \;S\;I_{1}-\beta \;S-\tau \;S \end{array}$$ (17)

$$\begin{array}{c} g_{2}=\phi \;\chi \;S\;I_{1}-\;\varsigma \;\;E-\tau \;E \end{array}$$ (18)

$$\begin{array}{c} g_{3}=\Phi \;\;\varsigma \;\;E-\tau \;I_{1}-\psi \;I_{1}-\alpha \;I_{1} \end{array}$$ (19)

$$\begin{array}{c} g_{4}=\alpha \;I_{1}+\psi (1-\omega )I_{1}-(\upsilon +\rho +\tau )I_{2} \end{array}$$ (20)

$$\begin{array}{c} g_{5}=(1-\Phi )\;\varsigma \;\;E+\upsilon \;I_{2}+\psi \;\omega \;I_{1}-(\tau +\lambda )R \end{array}$$ (21)

and

$$\begin{array}{c} g_{6}=\beta \;S+(1-\zeta )\lambda \;R\tau \;Q. \end{array}$$ (22)

We show that: $\frac{\partial g_{i}}{\partial x_{j}}$, i, j = 1, 2, 3, 4 are continuous and bounded. For each of the model equations, we investigated the following partial derivatives:

From Eq (17);

$$\{\begin{array}{l} \frac{\partial g_{1}}{\partial S}=-\phi \chi I_{1}-\left(\beta +\tau \right),|\frac{\partial g_{1}}{\partial S}|=|-\phi \chi I_{1}-\left(\beta +\tau \right)|<\infty \\ \frac{\partial g_{1}}{\partial E}=0,|\frac{\partial g_{1}}{\partial E}|=|0|<\infty \\ \frac{\partial g_{1}}{\partial I_{1}}=-\phi \chi I_{1},|\frac{\partial g_{1}}{\partial I_{1}}|=|-\phi \chi I_{1}|<\infty \\ \frac{\partial g_{1}}{\partial I_{2}}=0,|\frac{\partial g_{1}}{\partial I_{2}}|=|0|<\infty \\ \frac{\partial g_{1}}{\partial R}=\zeta \lambda ,|\frac{\partial g_{1}}{\partial R}|=|\zeta \lambda |<\infty \\ \frac{\partial g_{1}}{\partial Q}=0,|\frac{\partial g_{1}}{\partial Q}|=|0|<\infty . \end{array}$$ (23)

Similarly, from Eq (18) we also have that:

$$\{\begin{array}{l} \frac{\partial g_{2}}{\partial S}=-\phi \chi I_{1},|\frac{\partial g_{2}}{\partial S}|=|-\phi \chi I_{1}|<\infty \\ \frac{\partial g_{2}}{\partial E}=-(\;\varsigma \;+\tau ),|\frac{\partial g_{2}}{\partial E}|=|-(\;\varsigma \;+\tau )|<\infty \\ \frac{\partial g_{2}}{\partial I_{1}}=-\phi \chi S,|\frac{\partial g_{2}}{\partial I_{1}}|=|-\phi \chi S|<\infty \\ \frac{\partial g_{2}}{\partial I_{2}}=0,|\frac{\partial g_{2}}{\partial I_{2}}|=|0|<\infty \\ \frac{\partial g_{2}}{\partial R}=0,|\frac{\partial g_{2}}{\partial R}|=|0|<\infty \\ \frac{\partial g_{2}}{\partial Q}=0,|\frac{\partial g_{2}}{\partial Q}|=|0|<\infty . \end{array}$$ (24)

From Eq (19) we also have that:

$$\{\begin{array}{l} \frac{\partial g_{3}}{\partial S}=0,|\frac{\partial g_{3}}{\partial S}|=|0|<\infty \\ \frac{\partial g_{3}}{\partial E}=\Phi \;\varsigma \;,|\frac{\partial g_{3}}{\partial E}|=|\Phi \;\varsigma \;|<\infty \\ \frac{\partial g_{3}}{\partial I_{1}}=-(\tau +\psi +\alpha ),|\frac{\partial g_{3}}{\partial I_{1}}|=|-(\tau +\psi +\alpha )|<\infty \\ \frac{\partial g_{3}}{\partial I_{2}}=0,|\frac{\partial g_{3}}{\partial I_{2}}|=|0|<\infty \\ \frac{\partial g_{3}}{\partial R}=0,|\frac{\partial g_{3}}{\partial R}|=|0|<\infty \\ \frac{\partial g_{3}}{\partial Q}=0,|\frac{\partial g_{3}}{\partial Q}|=|0|<\infty . \end{array}$$ (25)

From Eq (20) we also have that:

$$\{\begin{array}{l} \frac{\partial g_{4}}{\partial S}=0,|\frac{\partial g_{4}}{\partial S}|=|0|<\infty \\ \frac{\partial g_{4}}{\partial E}=0,|\frac{\partial g_{4}}{\partial E}|=|0|<\infty \\ \frac{\partial g_{4}}{\partial I_{1}}=\alpha +\psi (1-\omega ),|\frac{\partial g_{4}}{\partial I_{1}}|=|\alpha +\psi (1-\omega )|<\infty \\ \frac{\partial g_{4}}{\partial I_{2}}=-(\upsilon +\rho +\tau ),|\frac{\partial g_{4}}{\partial I_{2}}|=|-(\upsilon +\rho +\tau )|<\infty \\ \frac{\partial g_{4}}{\partial R}=0,|\frac{\partial g_{4}}{\partial R}|=|0|<\infty \\ \frac{\partial g_{4}}{\partial Q}=0,|\frac{\partial g_{4}}{\partial Q}|=|0|<\infty . \end{array}$$ (26)

Also from Eq (21) we also have that:

$$\{\begin{array}{l} \frac{\partial g_{5}}{\partial S}=0,|\frac{\partial g_{5}}{\partial S}|=|0|<\infty \\ \frac{\partial g_{5}}{\partial E}=(1-\Phi )\;\varsigma \;,|\frac{\partial g_{5}}{\partial E}|=|(1-\Phi )\;\varsigma \;|<\infty \\ \frac{\partial g_{5}}{\partial I_{1}}=\psi \omega ,|\frac{\partial g_{5}}{\partial I_{1}}|=|\psi \omega |<\infty \\ \frac{\partial g_{5}}{\partial I_{2}}=\upsilon ,|\frac{\partial g_{5}}{\partial I_{2}}|=|\upsilon |<\infty \\ \frac{\partial g_{5}}{\partial R}=-(\tau +\lambda ),|\frac{\partial g_{5}}{\partial R}|=|-(\tau +\lambda )|<\infty \\ \frac{\partial g_{5}}{\partial Q}=0,|\frac{\partial g_{5}}{\partial Q}|=|0|<\infty . \end{array}$$ (27)

Finally from Eq (22) we have:

$$\{\begin{array}{l} \frac{\partial g_{6}}{\partial S}=\beta ,|\frac{\partial g_{6}}{\partial S}|=|\beta |<\infty \\ \frac{\partial g_{6}}{\partial E}=0,|\frac{\partial g_{6}}{\partial E}|=|0|<\infty \\ \frac{\partial g_{6}}{\partial I_{1}}=0,|\frac{\partial g_{6}}{\partial I_{1}}|=|0|<\infty \\ \frac{\partial g_{6}}{\partial I_{2}}=0,|\frac{\partial g_{6}}{\partial I_{2}}|=|0|<\infty \\ \frac{\partial g_{6}}{\partial R}=(1-\zeta )\lambda ,|\frac{\partial g_{6}}{\partial R}|=|(1-\zeta )\lambda |<\infty \\ \frac{\partial g_{6}}{\partial Q}=-\tau ,|\frac{\partial g_{6}}{\partial Q}|=|-\tau |<\infty . \end{array}$$ (28)

We have successfully shown that each of these partial derivatives is continuous and bounded, thus we can conclude from the theorem Uniqueness of SMAD Model’s Solution that there is only one solution to the model (1) in the domain Ω.

## 6 Stability analysis and bifurcation of the steady states of SMAD model

We will define the stability and bifurcation analysis of the SMAD model equilibrium points in this section. All the equilibrium states of SMAD are given as:

Disease-free equilibrium point (DFE):

$$\begin{array}{c} E_{q0}=(S^{0},E^{0},{I_{1}}^{0},{I_{2}}^{0},R^{0},Q^{0}). \end{array}$$

Endemic equilibrium point (EEP):

$$\begin{array}{c} E_{q1}=(S^{1},E^{1},{I_{1}}^{1},{I_{2}}^{1},R^{1},Q^{1}). \end{array}$$

The thresh hold parameter **R**_0_ of SMAD is given as

$$\begin{array}{c} R_{0}\left(E,I_{1},I_{2},R\right)=\frac{\Lambda \;\;\varsigma \;\;\phi \;\chi \;\Phi }{\left(\alpha +\psi +\tau )\;(\beta +\tau )\;(\;\varsigma \;+\tau \right)}\;\;\;where\;\;\left(\alpha +\psi +\tau \right)\;\left(\beta +\tau \right)\;\left(\;\varsigma \;+\tau \right)\neq 0 \end{array}$$

Epidemiologically,

- if **R**_0_ < 1, the occurrence of the disease will decrease.
- if **R**_0_ = 1, the disease occurrence will be constant.
- if **R**_0_ > 1 the occurrence of the disease will increase.

In view of these remarks on our model, we have

- if Λ *ς* ϕ *χ* Φ < (*α* + *ψ* + *τ*) (*β* + *τ*) (*ς* + *τ*), the occurrence of the addiction and depression will decrease.
- if Λ *ς* ϕ *χ* Φ = (*α* + *ψ* + *τ*) (*β* + *τ*) (*ς* + *τ*), the addiction and depression occurrence will be constant.
- if Λ *ς* ϕ *χ* Φ > (*α* + *ψ* + *τ*) (*β* + *τ*) (*ς* + *τ*) the occurrence of the addiction and depression will increase.

Thus, we have also determined the following conclusion.

### 6.1 Stability analysis of disease Free equilibrium point (*E*_*q*0_)

#### 6.1.1 Local stability of SMADDFE

**Theorem**

The disease-free equilibrium point is locally asymptotically stable if **R**_0_ < 1 and unstable if **R**_0_ > 1.

**Proof**

The Jacobian of the system (1) is given by following formula:

$$\begin{array}{c} J\left(g_{i},i=1,2,3,4,5,6\right)=(\begin{array}{cccccc} \frac{\partial g_{1}}{\partial S} & \frac{\partial g_{1}}{\partial E} & \frac{\partial g_{1}}{\partial I_{1}} & \frac{\partial g_{1}}{\partial I_{2}} & \frac{\partial g_{1}}{\partial R} & \frac{\partial g_{1}}{\partial Q}\\ \frac{\partial g_{2}}{\partial S} & \frac{\partial g_{2}}{\partial E} & \frac{\partial g_{2}}{\partial I_{1}} & \frac{\partial g_{2}}{\partial I_{2}} & \frac{\partial g_{2}}{\partial R} & \frac{\partial g_{2}}{\partial Q}\\ \frac{\partial g_{3}}{\partial S} & \frac{\partial g_{3}}{\partial E} & \frac{\partial g_{3}}{\partial I_{1}} & \frac{\partial g_{3}}{\partial I_{2}} & \frac{\partial g_{3}}{\partial R} & \frac{\partial g_{3}}{\partial Q}\\ \frac{\partial g_{4}}{\partial S} & \frac{\partial g_{4}}{\partial E} & \frac{\partial g_{4}}{\partial I_{1}} & \frac{\partial g_{4}}{\partial I_{2}} & \frac{\partial g_{4}}{\partial R} & \frac{\partial g_{4}}{\partial Q}\\ \frac{\partial g_{5}}{\partial S} & \frac{\partial g_{5}}{\partial E} & \frac{\partial g_{5}}{\partial I_{1}} & \frac{\partial g_{5}}{\partial I_{2}} & \frac{\partial g_{5}}{\partial R} & \frac{\partial g_{5}}{\partial Q}\\ \frac{\partial g_{6}}{\partial S} & \frac{\partial g_{6}}{\partial E} & \frac{\partial g_{6}}{\partial I_{1}} & \frac{\partial g_{6}}{\partial I_{2}} & \frac{\partial g_{6}}{\partial R} & \frac{\partial g_{6}}{\partial Q} \end{array}) \end{array}$$

So that;

$$\begin{array}{c} J=(\begin{array}{cccccc} -\phi \;\chi \;A-\beta -\tau & 0 & -\phi \;\chi \;S & 0 & \zeta \;\lambda & 0\\ \phi \;\chi \;A & -\;\varsigma \;-\tau & \phi \;\chi \;S & 0 & 0 & 0\\ 0 & \Phi \;\;\varsigma \; & -\tau -\alpha -\psi & 0 & 0 & 0\\ 0 & 0 & \alpha +\psi \;(1-\omega ) & -\upsilon -\rho -\tau & 0 & 0\\ 0 & (1-\Phi )\;\varsigma \; & \omega \;\psi & \upsilon & -\tau -\lambda & 0\\ \beta & 0 & 0 & 0 & (1-\zeta )\lambda & -\tau \end{array}) \end{array}$$ (29)

Simulating Eq (29) at the SMADDFE $E_{q0}=\left(S^{0},E^{0},{I_{1}}^{0},{I_{2}}^{0},R^{0},Q^{0}\right)$, we get:

$$\begin{array}{c} J\left(E_{q0}\right)=(\begin{array}{cccccc} -\beta -\tau & 0 & -\frac{\phi \;\chi \;\Lambda }{\beta +\tau } & 0 & \zeta \;\lambda & 0\\ 0 & -\;\varsigma \;-\tau & \frac{\phi \;\chi \;\Lambda }{\beta +\tau } & 0 & 0 & 0\\ 0 & \Phi \;\;\varsigma \; & -\tau -\alpha -\psi & 0 & 0 & 0\\ 0 & 0 & -\omega \;\psi +\alpha +\psi & -\upsilon -\rho -\tau & 0 & 0\\ 0 & -(-1+\Phi )\;\varsigma \; & \omega \;\psi & \upsilon & -\tau -\lambda & 0\\ \beta & 0 & 0 & 0 & -(-1+\zeta )\lambda & -\tau \end{array}) \end{array}$$ (30)

The eigenvalues are given by converting (30) into block matrices such as:

$$\begin{array}{c} |J(E_{q0}-I\lambda_{1}|=0 \end{array}$$ (31)

Thus;

$$\begin{array}{c} \left|J(E_{q0}-I\lambda_{1}|=|\begin{array}{cc} M_{11}-I\lambda_{1} & M_{12}\\ M_{21} & M_{22}-I\lambda_{1} \end{array}\right| \end{array}$$ (32)

Where

$$\begin{array}{c} M_{11}-I\lambda_{1}=(\begin{array}{ccc} -\beta -\tau -\lambda_{1} & 0 & -\frac{\phi \;\chi \;\Lambda }{\beta +\tau }\\ 0 & -\;\varsigma \;-\tau -\lambda_{1} & \frac{\phi \;\chi \;\Lambda }{\beta +\tau }\\ 0 & \Phi \;\;\varsigma \; & -\tau -\alpha -\psi -\lambda_{1} \end{array}) \end{array}$$

$$\begin{array}{c} M_{12}=(\begin{array}{ccc} 0 & \zeta \;\lambda & 0\\ 0 & 0 & 0\\ 0 & 0 & 0 \end{array}) \end{array}$$

$$\begin{array}{c} M_{21}=(\begin{array}{ccc} 0 & 0 & -\omega \;\psi +\alpha \;+\psi \\ 0 & -(-1+\Phi )\;\varsigma \; & \omega \;\psi \\ \beta & 0 & 0 \end{array}) \end{array}$$

$$\begin{array}{c} M_{22}-I\lambda_{1}=(\begin{array}{ccc} -\upsilon -\rho -\tau -\lambda_{1} & 0 & 0\\ \upsilon & -\tau -\upsilon -\lambda_{1} & 0\\ 0 & -(-1+\zeta )\lambda & -\tau -\lambda_{1} \end{array}) \end{array}$$

calculate values

$$\{\begin{array}{l} |M_{11}-I\lambda_{1}|=(-\beta -\tau -\lambda_{1})((-\;\varsigma \;-\tau -\lambda_{1})(-\tau -\alpha -\psi -\lambda_{1})-\frac{\Phi \;\varsigma \;\phi \chi \Lambda }{\beta +\tau }),\\ |M_{12}|=0,\\ |M_{21}|=-\beta (1-\psi )\;\varsigma \;(-\omega \psi +\alpha +\psi ),\\ |M_{22}-I\lambda_{1}|=(-\upsilon -\rho -\tau -\lambda_{1})(-\tau -\upsilon -\lambda_{1})(-\tau -\lambda_{1}). \end{array}$$

Putting values of |*M*_11_ − *I*λ_1_|, |*M*_12_|, |*M*_21_|*and*|*M*_22_ − *I*λ_1_| in (32), the characteristics equations as follows:

$$\begin{array}{c} \left(-\upsilon -\rho -\tau -\lambda_{1}\right)\left(-\tau -\upsilon -\lambda_{1}\right)\left(-\tau -\lambda_{1}\right)\left(-\beta -\tau -\lambda_{1}\right)\\ \left(\left(-\;\varsigma \;-\tau -\lambda_{1}\right)\left(-\tau -\alpha -\psi -\lambda_{1}\right)-\frac{\Phi \;\;\varsigma \;\;\phi \;\chi \;\Lambda }{\beta +\tau }\right)=0 \end{array}$$

From this equation the four eigenvalues are

$$\begin{array}{c} -(\upsilon +\rho +\tau ),-(\tau +\upsilon ),-\tau ,-(\beta +\tau ) \end{array}$$

Using the following quadratic equation, the other eigenvalues are determined.

$$\begin{array}{c} \lambda_{1}^{2}+a_{1}\lambda_{1}+a_{2}=0 \end{array}$$ (33)

where

$$\begin{array}{c} a_{1}=\;\varsigma \;+\alpha +\psi +2\mu ,\\ a_{2}=\left(\;\varsigma \;+\tau \right)\left(\alpha +\psi +\tau \right)-\frac{\Phi \;\;\varsigma \;\;\phi \;\chi \;\Lambda }{\beta +\tau }. \end{array}$$

Routh-Hurwitz criteria were employed to determine the two roots’ negativity and according to the criterion, the real root of Eq (33) is strictly negative. if and only if *a*_1_ > 0, *a*_2_ > 0 and *a*_1_*a*_2_ > 0. It is evident that *a*_1_ > 0 and *a*_2_ can be shown as

$$\begin{array}{c} a_{2}=\left(\;\varsigma \;+\tau \right)\left(\alpha +\psi +\tau \right)-\frac{\Phi \;\;\varsigma \;\;\phi \;\chi \;\Lambda }{\beta +\tau }.\\ a_{2}=\left(\;\varsigma \;+\tau \right)\left(\alpha +\psi +\tau \right)\left(1-\frac{\Phi \;\;\varsigma \;\;\phi \;\chi \;\Lambda }{\left(\beta +\tau )(\;\varsigma \;+\tau )(\alpha +\psi +\tau \right)}\right)\\ a_{2}=\left(\;\varsigma \;+\tau \right)\left(\alpha +\psi +\tau \right)\left(1-R_{0}\right). \end{array}$$

Hence all eigenvalues are negative, (33) satisfy the Routh-Hurwitz criteria, and also **R**_0_ < 1 condition holds. Therefore DFE is locally asymptotically stable.

#### 6.1.2 Global stability of SMADDFE

In this subsection, we identify several fundamental concepts and notations for the consideration of the system’s global stability, which includes the DFE of SMAD. The Castillo-Chavez method would be used to prove that model (1) is globally asymptotically stable at the DFE [42, 46, 48, 49].

**Notation**

Let a matrix *J* > 0(< 0), if J is symmetric positive definite (or symmetric negative definite). Then, the following basic assumptions on matrix stability holds

**Lemma**

Let J be a *n* × *n* real matrix. Then all λ_*i*_ of J have negative (positive) real parts iff ∃ a matrix *H* > 0 such that *HJ* + *J*^*T*^*H*^*T*^ < 0(> 0).

**Lemma**

Assume a disease model system with the following structure:

$$\left\{ \begin{array}{l} \frac{\text{d}s_{1}}{\text{d}t}=x_{1}(s_{1},s_{2})\\ \frac{\text{d}s_{2}}{\text{d}t}=x_{2}(s_{1},s_{2}),x_{2}(s_{1},0)=0. \end{array}\right.$$ (34)

where $s_{1}\in {ℜ}^{m}$ denotes the uninfected population and $s_{2}\in {ℜ}^{n}$ represents the infected. $s^{*}=\left(s_{1}^{0},0\right)$ is the DFE of the given system (34).

For the specified system, the point $\left(x_{1}^{0},0\right)$ is a globally asymptotically stable equilibrium provided **R**_0_ < 1, and the following conditions are satisfied.

**B**1: For $\frac{\text{d}s_{1}}{\text{d}t}=x_{1}\left(s_{1},0\right),s_{1}^{0}$ is a globally asymptotically stable.
**B**2: $\frac{\text{d}s_{2}}{\text{d}t}=J\;s_{2}-\hat{x_{2}}\left(s_{1},s_{2}\right)$ with $\hat{x_{2}}\left(s_{1},s_{2}\right)\geq 0$ for (*s*_1_, *s*_2_) ∈ Π where $J=\frac{\partial x_{2}}{\partial t}\left(x_{1}\left(s_{1}^{0},0\right)\right)$ is the Jacobi matrix which contains all non-negative off-diagonal components (Metzler matrix) and Π is the region where the model makes biological sense.

**Lemma**

Let $S=|\begin{array}{cc} s_{11} & s_{12}\\ s_{21} & s_{22} \end{array}|$ be the *n* × *m* matrix where n = m = 2. Then S is stable if an only if *s*_11_ < 0, *s*_22_ < 0 and |*S*| > 0. This explain by the following definition.

**Definition**

A non-singular *n* × *n* matrix S is diagonally stable (or positive stable) if *a* positive diagonal *n* × *n* matrix R such that *RS* + *S*^*T*^*R*^*T*^ > 0.

**Theorem(Krasovkil LaSalle theorem)**

Suppose the autonomous system *x*_0_ = *N*(*x*), where x* is a steady state, that is *N*(x*) = 0. Assume, ∃ continuously differentiable function *f*: ℜ^*n*^ → ℜ and that this function is radially unbounded and positive definite across the entire space and that is satisfies

$$\begin{array}{c} f^{\prime }\left(x\right)\leq 0\;\;\;\;\;\forall \;\;t\;\;\;\;and\;\;\;\;\;\forall \;\;x\in {ℜ}^{n} \end{array}$$

Define the invariant set

$$\begin{array}{c} T=\{x\in {ℜ}^{n}|f^{\prime }\left(x\right)=0\}. \end{array}$$

If T contains only the equilibrium x* is globally stable.

**Theorem(Global stability of DFE)**

The DFE *E*_*q*0_ = (*S*^0^, *E*^0^, *I*_1_^0^, *I*_2_^0^, *R*^0^, *Q*^0^) is the global asymptotically stable equilibrium of the system (1) provided that ℜ<1 and the assumption of Eq (34) satisfied.

**Proof**

Suppose for model (1) the uninfected population is $s_{1}=\left(S,Q\right)\in {ℜ}^{3}$ and infected population is $s_{1}=\left(E,I_{1},I_{2},R\right)\in {ℜ}^{3}$. From model (1), we write *x*_1_(*s*_1_, *s*_2_) and *x*_2_(*s*_1_, *s*_2_) as

$$\left\{ \begin{array}{l} \frac{\text{d}S}{\text{d}t}=\Lambda +\zeta \lambda R-\phi \chi SI_{1}-\beta S-\tau S,\\ \frac{\text{d}Q}{\text{d}t}=\beta S+\left(1-\zeta \right)\lambda R-\tau Q. \end{array}\right.$$ (35)

Thus, we have

$$\begin{array}{c} x_{1}\left(s_{1},s_{2}\right)=\left(\begin{array}{l} \Lambda +\zeta \lambda R-\phi \chi I_{1}S-(\beta +\tau )S,\\ \beta S+\left(1-\zeta \right)\lambda R-\tau Q. \end{array}\right) \end{array}$$ (36)

At $s^{*}=\left(s_{1}^{0},0\right)$, Eq (36) becomes

$$\begin{array}{c} \frac{\text{d}s_{1}}{\text{d}t}=x_{1}\left(s_{1},0\right)=\left(\begin{array}{l} \Lambda +\zeta \lambda R-\phi \chi SI_{1}-\beta S-\tau S,\\ \beta S+\left(1-\zeta \right)\lambda R-\tau Q. \end{array}\right) \end{array}$$ (37)

$$\begin{array}{c} \frac{\text{d}s_{1}}{\text{d}t}=x_{1}\left(s_{1},0\right)=(\begin{array}{c} \Lambda -(\beta +\tau )S\\ \beta \;S-\tau \;Q \end{array}) \end{array}$$ (38)

From Eq (38), we have

$$\{\begin{array}{l} S\left(t\right)=S\left(0\right)e^{-\left(\beta +\tau \right)t}+\frac{\Lambda }{\beta +\tau }\left(1-e^{-\left(\beta +\tau \right)t}\right)\\ Q\left(t\right)=Q\left(0\right)e^{-\tau \;t}+\frac{\beta \Lambda }{(\beta +\tau )\tau }\left(1-e^{-\tau \;t}\right) \end{array}$$ (39)

As *t* → ∞ the solution is:

$$\{\begin{array}{l} S\left(\infty \right)=\frac{\Lambda }{\beta +\tau }\\ Q\left(\infty \right)=\frac{\beta \Lambda }{(\beta +\tau )\tau }. \end{array}$$ (40)

which implies the global convergence of (38) in Π, and this satisfies condition **B1**.

Now by (1), we have

$$\{\begin{array}{l} \frac{\text{d}E}{\text{d}t}=\phi \chi SI_{1}-\;\varsigma \;E-\tau E,\\ \frac{\text{d}I_{1}}{\text{d}t}=\Phi \;\varsigma \;E-\tau I_{1}-\psi I_{1}-\alpha I_{1},\\ \frac{\text{d}I_{2}}{\text{d}t}=\alpha I_{1}+\psi \left(1-\omega \right)I_{1}-\left(\upsilon +\rho +\tau \right)I_{2},\\ \frac{\text{d}R}{\text{d}t}=\left(1-\Phi \right)\;\varsigma \;E+\upsilon I_{2}+\psi \omega I_{1}-\left(\tau +\lambda \right)R \end{array}$$ (41)

$$\begin{array}{c} \frac{\text{d}s_{2}}{\text{d}t}=x_{2}\left(s_{1},s_{2}\right)=\left(\begin{array}{l} \phi \chi SI_{1}-\;\varsigma \;E-\tau E,\\ \Phi \;\varsigma \;E-\tau I_{1}-\psi I_{1}-\alpha I_{1},\\ \alpha I_{1}+\psi \left(1-\omega \right)I_{1}-\left(\upsilon +\rho +\tau \right)I_{2},\\ \left(1-\Phi \right)\;\varsigma \;E+\upsilon I_{2}+\psi \omega I_{1}-\left(\tau +\lambda \right)R. \end{array}\right) \end{array}$$ (42)

Now we compute

$$\begin{array}{c} J=(\begin{array}{cccc} -(\;\varsigma \;+\tau ) & \frac{\phi \;\chi \;\Lambda }{\left(\beta +\tau \right)} & 0 & 0\\ \Phi \;\;\varsigma \; & -(\tau +\psi +\alpha ) & 0 & 0\\ 0 & \alpha +\psi (1-\omega ) & -(\upsilon +\rho +\tau ) & 0\\ (1-\Phi )\;\varsigma \; & \psi \;\omega & \upsilon & \left(\tau +\lambda \right) \end{array}) \end{array}$$ (43)

$$\begin{array}{c} s_{2}=(\begin{array}{c} E\\ I_{1}\\ I_{2}\\ R \end{array}) \end{array}$$ (44)

$$\begin{array}{c} J\;s_{2}=\left(\begin{array}{l} -(\;\varsigma \;+\tau )E+\frac{\phi \chi \Lambda }{\left(\beta +\tau \right)}I_{1}\\ \Phi \;\varsigma \;E-(\tau +\psi +\alpha )I_{1}\\ (\alpha +\psi (1-\omega ))I_{1}-(\upsilon +\rho +\tau )I_{2}\\ (1-\Phi )\;\varsigma \;E+\psi \omega I_{1}+\upsilon I_{2}+(\tau +\lambda )R \end{array}\right) \end{array}$$ (45)

$$\begin{array}{c} \hat{x_{2}}\left(s_{1},s_{2}\right)=(\begin{array}{c} \phi \;\chi \;I_{1}\left(S^{0}-S\right)\\ 0\\ 0\\ 0 \end{array}) \end{array}$$ (46)

Here, since S* = *S*^0^ ≥ *S*. It is clear that $\hat{x_{2}}\left(s_{1},s_{2}\right)\geq 0$ for all (*s*_1_, *s*_2_) ∈ Π. Now putting Eqs (42)–(46) in condition **B2**, we have

$$\begin{array}{c} \frac{\text{d}s_{2}}{\text{d}t}=J\;s_{2}-\hat{x_{2}}\left(s_{1},s_{2}\right)\;\;\;with\;\;\;\;\;\hat{x_{2}}\left(s_{1},s_{2}\right)\geq 0 \end{array}$$

$$\begin{array}{c} \frac{\text{d}s_{2}}{\text{d}t}=(\begin{array}{cccc} -(\;\varsigma \;+\tau ) & \frac{\phi \;\chi \;\Lambda }{\left(\beta +\tau \right)} & 0 & 0\\ \Phi \;\;\varsigma \; & -(\tau +\psi +\alpha ) & 0 & 0\\ 0 & \alpha +\psi (1-\omega ) & -(\upsilon +\rho +\tau ) & 0\\ (1-\Phi )\;\varsigma \; & \psi \;\omega & \upsilon & \left(\tau +\lambda \right) \end{array})\;\left(\begin{array}{c} E\\ I_{1}\\ I_{2}\\ R \end{array}\right)-\left(\begin{array}{c} \theta \\ 0\\ 0\\ 0 \end{array}\right) \end{array}$$

where *θ* = *ϕ χ I*_1_(*S*^0^ − *S*). Hence, it indicates that SMADDFE is globally asymptotically stable.

### 6.2 Stability and bifurcation analysis of endemic equilibrium point (*E*_*q*1_)

The short-term breakouts of the disease are caused by the stability of disease-free equilibrium points. Over a longer time, its dynamics are characterized by stability at the endemic equilibrium sites. We discovered that persistent behavior influences critical epidemiological results, including whether an outbreak of a disease would remain in an endemic condition or if the disease will disperse. We will conduct a stability analysis of SMADEEP here. For analyses of the condition of the bifurcation and the local stability of EEP, we utilized the further approach provided by Castillo Chavez and Song [42, 46, 48, 49].

#### 6.2.1 Local stability analysis of SMADEEP

**Theorem**(**Lyapunov’s theorem**)

For an autonomous system of ordinary differential equations of the form

$$\begin{array}{c} \frac{\text{d}z}{\text{d}t}=f\left(z\right) \end{array}$$

where z is a vector in n-dimensional space, if ∃ a continuously differentiable function *T*(*z*) such that:

- T(z) is positive definite such that
  $$\begin{array}{c} T(z)>0\;\;\forall \;\;z\neq 0\;\;\;\;and\;\;\;T(0)=0. \end{array}$$
- The derivative of T(z) along the solutions of the system is negative semi-definite such that
  $$\begin{array}{c} \frac{\text{d}T(z)}{\text{d}t}\leq 0\;\;\forall \;\;x. \end{array}$$
-
$\frac{\text{d}T(z)}{\text{d}t}=0$ only at the equilibrium point(s) of the system.

Then the equilibrium point(s) of the system are Lyapunov stable. If $\frac{\text{d}T(z)}{\text{d}t}<0$ ∀ *x* ≠ 0, then the equilibrium point(s) of the system are asymptotically stable [46].

**Theorem**

If **R**_0_ > 1, then the endemic equilibrium *E*_*q*1_ of system (1) is locally asymptotically stable.

**Proof**

By using the Lyapunov’s theorem, We have Lyapunov’s function

$$\begin{array}{c} L=\frac{1}{2}(S^{2}+E^{2}+I_{1}^{2}+I_{2}^{2}+R^{2}+Q^{2}) \end{array}$$

This Lyapunov’s function is positive definite. Now, we take the derivative of Lyapunov’s function with respect to t.

$$\begin{array}{c} \frac{\text{d}L}{\text{d}t}=\frac{1}{2}(\frac{\text{d}S^{2}}{\text{d}t}+\frac{\text{d}E^{2}}{\text{d}t}+\frac{\text{d}I_{1}^{2}}{\text{d}t}+\frac{\text{d}I_{2}^{2}}{\text{d}t}+\frac{\text{d}R^{2}}{\text{d}t}+\frac{\text{d}Q^{2}}{\text{d}t}) \end{array}$$

$$\begin{array}{c} \frac{\text{d}L}{\text{d}t}=\frac{1}{2}(2S\frac{\text{d}S}{\text{d}t}+2E\frac{\text{d}E}{\text{d}t}+2I_{1}\frac{\text{d}I_{1}}{\text{d}t}+2I_{2}\frac{\text{d}I_{2}}{\text{d}t}+2R\frac{\text{d}R}{\text{d}t}+2Q\frac{\text{d}Q}{\text{d}t}) \end{array}$$

Putting values of derivatives by using model (1), we get

$$\begin{array}{c} \frac{\text{d}L}{\text{d}t}=\{\begin{array}{l} S(\Lambda +\zeta \lambda R-\phi \chi SI_{1}-\beta S-\tau ,S)+E(\phi \chi I_{1}S-\;\varsigma \;E-\tau E)+\\ I_{1}(\Phi \;\varsigma \;E-\tau I_{1}-\psi I_{1}-\alpha I_{1})+I_{2}(\alpha I_{1}+\psi \left(1-\omega \right)I_{1}-\upsilon I_{2}-\rho I_{2}-\tau I_{2})+\\ R(\left(1-\Phi \right)\;\varsigma \;E+\upsilon I_{2}+\psi \omega I_{1}-\tau R-\lambda R)+Q(\beta S+\left(1-\zeta \right)\lambda R-\tau Q). \end{array} \end{array}$$

By solving this, we get

$$\begin{array}{c} \frac{\text{d}L}{\text{d}t}=\{\begin{array}{l} -(\phi \chi I_{1}S^{2}+\left(\beta +\tau \right)S^{2}+\left(\;\varsigma \;+\tau \right)E^{2}+\left(\tau +\psi +\alpha \right)I_{1}^{2}+\left(\upsilon +\rho +\tau \right)I_{2}^{2}+\\ (\tau +\lambda )R^{2}+\tau Q^{2})+(S(\Lambda +\zeta \lambda R)+\phi \chi EI_{1}S+\Phi \;\varsigma \;I_{1}E+I_{2}(\alpha I_{1}+\\ \psi \left(1-\omega \right)I_{1})+R(\left(1-\Phi \right)\;\varsigma \;E+\upsilon I_{2}+\psi \omega I_{1})+Q(\beta S+\left(1-\zeta \right)\lambda R)) \end{array} \end{array}$$

From model (1), we know that Λ, *ζ*, *χ*, *ϕ*, *ς*, Φ, *α*, *ψ*, *υ*, *ρ*, *β*, *ω*, *τ* and λ are all non-negative, and the values of *S*, *E*, *I*_1_, *I*_2_, *R*, and *Q* are all non-negative. Therefore, we can see that all the terms inside the square brackets are non-negative, and the terms outside the square brackets are all non-positive. So, we have

$$-(\phi \;\chi \;I_{1}S^{2}+(\beta +\tau )S^{2}+(\;\varsigma \;+\tau )E^{2}+(\tau +\psi +\alpha )I_{1}^{2}+(\upsilon +\rho +\tau )I_{2}^{2}+(\tau +\lambda )R^{2}+\tau \;Q^{2})\leq 0$$

$$\begin{array}{c} (S\left(\Lambda +\zeta \;\lambda \;R\right)+\phi \;\chi \;E\;I_{1}\;S+\Phi \;\;\varsigma \;\;I_{1}E+I_{2}\left(\alpha \;I_{1}+\psi (1-\omega )I_{1}\right)\\ +R\left((1-\Phi )\;\varsigma \;\;E+\upsilon \;I_{2}+\psi \;\omega \;I_{1}\right)+Q\left(\beta \;S+(1-\zeta )\lambda \;R\right))\geq 0 \end{array}$$

Therefore, we can conclude that $\frac{\text{d}L}{\text{d}t}\leq 0$ for all non-zero vectors *S*, *E*, *I*_1_, *I*_2_, *R*,*Q* and non negative parameters that satisfy the model (1). Since the derivative of the Lyapunov function L is non-positive, and it is zero only at the origin *E*_*q*1_ = (*S*^1^, *E*^1^, *I*_1_^1^, *I*_2_^1^, *R*^1^, *Q*^1^), we can conclude that the origin is locally asymptotically stable.

#### 6.2.2 Global stability analysis of SMADEEP

**Theorem**

If **R**_0_ > 1, then the endemic equilibrium *E*_*q*1_ of system (1) is globally stable.

**Proof**

To carry out this study we use LaSalle’s invariant principle to define a Lyapunov function [50]

$$\begin{array}{c} G=(S-S^{1}-S^{1}log\frac{S^{1}}{S})+(E-E^{1}-E^{1}log\frac{E^{1}}{E})+(I_{1}-I_{1}^{1}-I_{1}^{1}log\frac{I_{1}^{1}}{I_{1}})+\\ \left(I_{2}-I_{2}^{1}-I_{2}^{1}log\frac{I_{2}^{1}}{I_{2}})+(R-R^{1}-R^{1}log\frac{R^{1}}{R})+(Q-Q^{1}-Q^{1}log\frac{Q^{1}}{Q}\right) \end{array}$$

$$\begin{array}{c} \frac{dG}{dt}=\left(\frac{S+S^{1}}{S}\right)S+\left(\frac{E+E^{1}}{E}\right)E+\left(\frac{I_{1}+I_{1}^{1}}{I_{1}}\right)I_{1}+\left(\frac{I_{2}+I_{2}^{1}}{I_{2}}\right)I_{2}+\left(\frac{R+R^{1}}{R}\right)R+\left(\frac{Q+Q^{1}}{Q}\right)Q\\ =(1+\frac{S^{1}}{S})(\Lambda +\zeta \;\lambda \;R-\phi \chi \;I_{1}\;S-\left(\beta +\tau \right)\rho )+(1+\frac{E^{1}}{E})(\phi \chi \;I_{1}\;S-\left(\;\varsigma \;+\tau \right)\;E)+\\ (1+\frac{I_{1}^{1}}{I_{1}})\left(\Phi \;\varsigma \;E-\left(\tau +\psi +\alpha \right)I_{1}\right)+(1+\frac{I_{2}^{1}}{I_{2}})\left(\alpha I_{1}+\psi \left(1-\omega \right)I_{1}-\left(\upsilon +\rho +\tau \right)I_{2}\right)+\\ (1+\frac{R^{1}}{R})\left(\left(1-\Phi \right)\;\varsigma \;E+\upsilon I_{2}+\psi \omega I_{1}-\left(\tau +\lambda \right)R\right)+(1+\frac{Q^{\prime }}{Q})\left(\beta S+\left(1-\zeta \right)\lambda R-\tau Q\right) \end{array}$$

$$\begin{array}{c} \frac{dG}{dt}=(1+\frac{S^{1}}{S})\left(\Lambda -\left(\beta +\tau \right)\left(S+S^{1}\right)+\zeta \lambda \left(R+R^{1}\right)\right)-(1+\frac{S^{\prime }}{S})\phi \chi \left(I_{1}+I_{1}^{1}\right)\left(S+S^{1}\right)+\\ \left(1+\frac{E^{1}}{E})\left(\;\varsigma \;+\tau \right)\left(E+E^{1}\right)+(1+\frac{E^{1}}{E})\left(\phi \chi \left(I_{1}+I_{1}^{1}\right)\left(S+S^{1}\right)\right)-(1+\frac{I_{1}^{1}}{I_{1}}\right)\\ \left(\tau +\psi +\alpha \right)\left(I_{1}+I_{1}^{1}\right)+(1+\frac{I_{1}^{1}}{I_{1}})\Phi \;\varsigma \;\left(E+E^{1}\right)-(1+\frac{I_{2}^{1}}{I_{2}})\left(\upsilon +\rho +\tau \right)\left(I_{2}+I_{2}^{1}\right)+(1+\frac{I_{2}^{1}}{I_{2}})\\ \left(\alpha +\psi \left(1-\omega \right)\right)\left(I_{1}+I_{1}^{1}\right)-(1+\frac{R^{\prime }}{R})\left(\tau +\lambda \right)\left(R+R^{1}\right)+(1+\frac{R^{\prime }}{R})(\left(1-\Phi \right)\;\varsigma \;\left(E+E^{1}\right)+\\ \upsilon \left(I_{2}+I_{2}^{1}\right)+\psi \omega \left(I_{1}+I_{1}^{1}\right))-(1+\frac{Q^{1}}{Q})\tau \left(Q+Q^{1}\right)+(1+\frac{Q^{1}}{Q})\left(\beta \left(S+S^{1}\right)+\left(1-\zeta \right)\lambda \left(R+R^{1}\right)\right) \end{array}$$

Collectively positive and negative term such that $\frac{dG}{dt}=M-N$

$$\begin{array}{c} M=(1+\frac{S^{1}}{S})\left(\Lambda +\zeta \lambda \left(R+R^{1}\right)\right)+(1+\frac{E^{1}}{E})\left(I_{1}+I_{1}^{1}\right)\left(S+S^{1}\right)\phi \chi +(1+\frac{I_{1}^{1}}{I_{1}})\left(E+E^{1}\right)\Phi \;\varsigma \;+\\ (1+\frac{I_{1}^{1}}{I_{1}})\left(I_{1}+I_{1}^{1}\right)\left(\alpha +\psi \left(1-\omega \right)\right)+(1+\frac{R^{1}}{R})\left(E+E^{1}\right)\left(1-\Phi \right)\;\varsigma \;+(1+\frac{R^{1}}{R})\left(I_{2}+I_{2}^{1}\right)\\ \upsilon +(1+\frac{R^{1}}{R})\left(I_{1}+I_{1}^{1}\right)\psi \omega +(1+\frac{Q^{1}}{Q})\left(S+S^{1}\right)\beta +(1+\frac{Q^{1}}{Q})\left(R+R^{1}\right)\left(1-\zeta \right)\lambda . \end{array}$$

$$\begin{array}{c} N=\frac{{\left(S+S^{1}\right)}^{2}}{S}\left(\beta +\tau \right)+\frac{{\left(S+S^{1}\right)}^{2}}{S}{\left(I_{1}+I_{1}^{1}\right)}^{2}\phi \chi +\frac{{\left(E+E^{1}\right)}^{2}}{E}\left(\;\varsigma \;+\tau \right)+\frac{{\left(I_{1}+I_{1}^{1}\right)}^{2}}{I_{1}}(\tau +\psi +\\ \alpha )+\frac{{\left(I_{2}+I_{2}^{1}\right)}^{2}}{I_{2}}\left(\upsilon +\rho +\tau \right)+\frac{{\left(R+R^{1}\right)}^{2}}{R}\left(\tau +\lambda \right)+\frac{{\left(Q+Q^{1}\right)}^{2}}{Q}\left(\tau \right). \end{array}$$

When *M* < *N*, then $\frac{\text{d}G}{\text{d}t}\leq 0$ will be negative definite along the solution path of the system which while $\frac{\text{d}G}{\text{d}t}=0$ at the endemic equilibrium. This according to LaSalle’s invariant principle therefore implies that the SMAEEP is globally asymptomatically stable.

#### 6.2.3 Forward bifurcation analysis of SMAD model

**Theorem**

If **R**_0_ > 1, then the EEP *E*_*q*1_ of model (1) is locally asymptotically stable in feasible region Δ and the model (1) shows forward bifurcation at **R**_0_ = 1 [42, 46, 48, 49].

**Proof**

To use the approach, the model system (1) is simplified, and the following variables are changed. Let

$$\begin{array}{c} S=y_{1},E=y_{2},I_{1}=y_{3},I_{2}=y_{4},R=y_{5},Q=y_{6} \end{array}$$

so that

$$\begin{array}{c} N=y_{1}+y_{2}+y_{3}+y_{4}+y_{5}+y_{6} \end{array}$$

Moreover, the model (1) becomes $\frac{dY}{dt}={\left(g_{1},g_{2},g_{3},g_{4},g_{5},g_{6}\right)}^{T}$ when we apply the vector notation *Y* = (*y*_1_, *y*_2_, *y*_3_, *y*_4_, *y*_5_, *y*_6_)^*T*^.

$$\{\begin{array}{l} \frac{\text{d}y_{1}}{\text{d}t}=g_{1}=\Lambda +\zeta \lambda y_{5}-\phi \chi y_{3}y_{1}-\left(\beta +\tau \right)y_{1},\\ \frac{\text{d}y_{2}}{\text{d}t}=g_{2}=\phi \chi y_{3}y_{1}-\left(\;\varsigma \;+\tau \right)y_{2},\\ \frac{\text{d}y_{3}}{\text{d}t}=g_{3}=\Phi \;\varsigma \;y_{2}-\left(\tau +\psi +\alpha \right)y_{3},\\ \frac{\text{d}y_{4}}{\text{d}t}=g_{4}=\alpha y_{3}+\psi \left(1-\omega \right)y_{3}-\left(\upsilon +\rho +\tau \right)y_{4},\\ \frac{\text{d}y_{5}}{\text{d}t}=g_{5}=\left(1-\Phi \right)\;\varsigma \;y_{2}+\upsilon y_{4}+\psi \omega y_{3}-\left(\tau +\lambda \right)y_{5},\\ \frac{\text{d}y_{6}}{\text{d}t}=g_{6}=\beta y_{1}+\left(1-\zeta \right)\lambda y_{5}-\tau y_{6}. \end{array}$$ (47)

The Jacobian of the model (1) is given as

$$\begin{array}{c} J=(\begin{array}{cccccc} -\phi \;\chi \;I_{1}-\beta -\tau & 0 & -\phi \;\chi \;S & 0 & \zeta \;\lambda & 0\\ \phi \;\chi \;I_{1} & -\;\varsigma \;-\tau & \phi \;\chi \;S & 0 & 0 & 0\\ 0 & \Phi \;\;\varsigma \; & -\tau -\alpha -\psi & 0 & 0 & 0\\ 0 & 0 & \alpha +\psi \;(1-\omega ) & -\upsilon -\rho -\tau & 0 & 0\\ 0 & (1-\Phi )\;\varsigma \; & \omega \;\psi & \upsilon & -\tau -\lambda & 0\\ \beta & 0 & 0 & 0 & (1-\zeta )\lambda & -\tau \end{array}) \end{array}$$ (48)

Evaluating Eq (47) at the SMADEEP $E_{q0}=\left(S^{0}=y_{1}^{0},E^{0}=y_{2}^{0},{I_{1}}^{0}=y_{3}^{0},{I_{2}}^{0}=y_{4}^{0},R^{0}=y_{5}^{0},Q^{0}=y_{6}^{0}\right)$, we get:

$$\begin{array}{c} J\left(E_{q0}\right)=(\begin{array}{cccccc} -\beta -\tau & 0 & -\frac{\phi \;\chi \;\Lambda }{\beta +\tau } & 0 & \zeta \;\lambda & 0\\ 0 & -\;\varsigma \;-\tau & \frac{\phi \;\chi \;\Lambda }{\beta +\tau } & 0 & 0 & 0\\ 0 & \Phi \;\;\varsigma \; & -\tau -\alpha -\psi & 0 & 0 & 0\\ 0 & 0 & -\omega \;\psi +\alpha +\psi & -\upsilon -\rho -\tau & 0 & 0\\ 0 & -(-1+\Phi )\;\varsigma \; & \omega \;\psi & \upsilon & -\tau -\lambda & 0\\ \beta & 0 & 0 & 0 & -(-1+\zeta )\lambda & -\tau \end{array}) \end{array}$$ (49)

The threshold parameter $R_{0}$ of SMAD is specified as

$$\begin{array}{c} R_{0}=\frac{\Lambda \;\;\varsigma \;\;\phi \;\chi \;\Phi }{\left(\alpha +\psi +\tau )\;(\beta +\tau )\;(\;\varsigma \;+\tau \right)} \end{array}$$

We use *ϕ* as a bifurcation parameters implies that **R**_0_ = 1 iff

$$\begin{array}{c} \phi =\phi^{*}=\frac{\left(\alpha +\psi +\tau )\;(\beta +\tau )\;(\;\varsigma \;+\tau \right)}{\Lambda \;\;\varsigma \;\;\chi \;\Phi } \end{array}$$

There is a clear zero eigenvalue in the linearized system of the modified model system (47) with *ϕ* = ϕ* as the bifurcation parameter.

$$\begin{array}{c} J\left(\phi^{*}\right)=(\begin{array}{cccccc} -\beta -\tau & 0 & -\frac{\left(\;\varsigma \;+\tau )(\tau +\alpha +\psi \right)}{\Phi \;\;\varsigma \;} & 0 & \zeta \;\lambda & 0\\ 0 & -\;\varsigma \;-\tau & \frac{\left(\;\varsigma \;+\tau )(\tau +\alpha +\psi \right)}{\Phi \;\;\varsigma \;} & 0 & 0 & 0\\ 0 & \Phi \;\;\varsigma \; & -\tau -\alpha -\psi & 0 & 0 & 0\\ 0 & 0 & -\psi \;\omega +\alpha +\psi & -\upsilon -\rho -\tau & 0 & 0\\ 0 & -(-1+\Phi )\;\varsigma \; & \psi \;\omega & \upsilon & -\tau -\lambda & 0\\ \beta & 0 & 0 & 0 & -(-1+\zeta )\lambda & -\tau \end{array}) \end{array}$$ (50)

The eigenvalues are

$$\begin{array}{c} 0,-\tau ,-(\tau +\lambda ),-(\beta +\tau ),-(\alpha +\;\varsigma \;+2\;\tau +\psi ),-(\upsilon +\rho +\tau ) \end{array}$$

Next, we find the left and right eigenvector V, W respectively, both of which are associated with the zero eigenvalues of the Jacobian of (50) at (represented by *J*_ϕ*_) selected such that *J*(*E*_*q*0_) × *W* = 0 and *V* × *J*(*E*_*q*0_) = 0 with *V*.*W* = 1, where

$$\begin{array}{c} W=\left[w_{1},w_{2},w_{3},w_{4},w_{5},w_{6}\right]\;\;\;\;\;\;V=\left[v_{1},v_{2},v_{3},v_{4},v_{5},v_{6}\right] \end{array}$$

$$\begin{array}{c} J\left(E_{q0}\right)\times W_{i}=0 \end{array}$$

Where

$$\begin{array}{c} W_{i}=(\begin{array}{c} w_{1}\\ w_{2}\\ w_{3}\\ w_{4}\\ w_{5}\\ w_{6} \end{array}) \end{array}$$

$$\begin{array}{c} (\begin{array}{cccccc} -\beta -\tau & 0 & -\frac{\phi \;\chi \;\Lambda }{\beta +\tau } & 0 & \zeta \;\lambda & 0\\ 0 & -\;\varsigma \;-\tau & \frac{\phi \;\chi \;\Lambda }{\beta +\tau } & 0 & 0 & 0\\ 0 & \Phi \;\;\varsigma \; & -\tau -\alpha -\psi & 0 & 0 & 0\\ 0 & 0 & -\omega \;\psi +\alpha +\psi & -\upsilon -\rho -\tau & 0 & 0\\ 0 & -(-1+\Phi )\;\varsigma \; & \omega \;\psi & \upsilon & -\tau -\lambda & 0\\ \beta & 0 & 0 & 0 & -(-1+\zeta )\lambda & -\tau \end{array})\times W_{i} \end{array}$$

$$\begin{array}{c} =(\begin{array}{c} 0\\ 0\\ 0\\ 0\\ 0\\ 0 \end{array}) \end{array}$$

Thus, We have

$$\{\begin{array}{ll} (-\beta -\tau )w_{1}-\frac{\phi \chi \Lambda }{\beta +\tau }w_{3}+\zeta \lambda w_{5} & =0,\\ (-\;\varsigma \;-\tau )w_{2}+\frac{\phi \chi \Lambda }{\beta +\tau }w_{3} & =0,\\ \Phi \;\varsigma \;w_{2}+(-\tau -\alpha -\psi )w_{3} & =0,\\ (-\omega \psi +\alpha +\psi )w_{3}+(-\upsilon -\rho -\tau )w_{4} & =0,\\ \left(1-\Phi \right)\;\varsigma \;w_{2}+\omega \psi w_{3}+\upsilon w_{4}+(-\tau -\lambda )w_{5} & =0\\ \beta w_{1}+\left(1-\zeta \right)\lambda w_{5}-\tau w_{6} & =0. \end{array}$$ (51)

Solving Eq (51), We get

$$\begin{array}{c} w_{1}=(-((\left(\zeta \;(-1+\Phi )\tau^{3}-((-\beta -\rho +(\omega -1)\psi -\alpha -\upsilon )\Phi +\psi +\rho +\beta +\alpha +\upsilon \right)\\ \zeta \;\tau^{2}+((\left(\left((-\omega +1)\psi +\rho +\alpha +\upsilon \right)\beta -\left((\omega -1)\psi -\alpha \right)\rho \right)\Phi +\left(-\psi -\rho -\alpha -\upsilon \right)\beta -\\ \left(\upsilon +\rho \right)\left(\psi +\alpha \right))\zeta +\phi \;\chi \;\Lambda \;\Phi )\tau -\beta \;(\rho \;((\omega -1)\psi -\alpha )\Phi +(\upsilon +\rho )(\psi +\alpha ))\zeta +\chi \;\Lambda \;\Phi \;\phi \\ \left(\upsilon +\rho \right))\lambda +\tau \;\chi \;\Lambda \;\Phi \;\phi \;\left(\upsilon +\rho +\tau \right))\;\varsigma \;)/\left({\left(\beta +\tau \right)}^{2}(\tau +\alpha +\psi )(\upsilon +\rho +\tau )(\tau +\lambda )\right))w_{2}. \end{array}$$ (52)

$$\begin{array}{c} \begin{array}{c} w_{2}>0\;\;\;\;\left(can\;take\;any\;value\right) \end{array} \end{array}$$ (53)

$$\begin{array}{c} \begin{array}{c} w_{3}=\frac{\Phi \;\;\varsigma \;\;w_{2}}{\tau +\alpha +\psi } \end{array} \end{array}$$ (54)

$$\begin{array}{c} \begin{array}{c} w_{4}=\frac{(-\omega \;\psi +\alpha +\psi )\Phi \;\;\varsigma \;\;w_{2}}{\left(\tau +\alpha +\psi )(\upsilon +\rho +\tau \right)} \end{array} \end{array}$$ (55)

$$\begin{array}{cc} w_{5}= & \begin{array}{c} -((-1+\Phi )\tau^{2}+(((-\omega +1)\psi +\rho +\alpha +\upsilon )\Phi -\psi -\rho -\alpha -\upsilon )\tau \\ -\rho \;((\omega -1)\psi -\alpha )\Phi -(\upsilon +\rho )(\psi +\alpha ))\;\varsigma \;\;w_{2}/(\tau +\alpha +\psi )(\upsilon +\rho \\ +\tau )(\tau +\lambda ) \end{array} \end{array}$$ (56)

$$\begin{array}{cc} w_{6}= & \begin{array}{c} (((((-1+\Phi )(-1+\zeta )\tau^{4}+((-1+\Phi )(\zeta -2)\beta -((-\rho +(\omega -1)\psi -\alpha -\upsilon )\Phi \\ +\psi +\rho +\alpha +\upsilon )(-1+\zeta ))\tau^{3}+((1-\Phi )\beta^{2}-((-\rho +(\omega -1)\psi -\alpha -\upsilon )\Phi +\psi +\\ \rho +\alpha +\upsilon )\left(\zeta -2\right)\beta -(-1+\zeta )\left(\rho \;\left((\omega -1)\psi -\alpha \right)\Phi +(\upsilon +\rho )(\psi +\alpha )\right))\tau^{2}-\\ (\left(\left((-\omega +1)\psi +\rho +\alpha +\upsilon \right)\Phi -\psi -\rho -\alpha -\upsilon \right)\beta +(\left((\omega -1)\psi -\alpha \right)\left(\zeta -2\right)\rho +\\ \phi \;\chi \;\Lambda )\Phi +\left(\upsilon +\rho \right)\left(\psi +\alpha \right)\left(\zeta -2\right))\beta \;\tau -((-\rho \;((\omega -1)\psi -\alpha )\Phi -(\upsilon +\rho )(\psi +\\ \alpha ))\beta +\chi \;\Lambda \;\Phi \;\phi \;(\upsilon +\rho ))\beta )\lambda -\tau \;\chi \;\Lambda \;\Phi \;\phi \;\beta \;(\upsilon +\rho +\tau ))\;\varsigma \;)/({\left(\beta +\tau \right)}^{2}(\tau +\alpha +\\ \psi )(\upsilon +\rho +\tau )(\tau +\lambda )\tau ))w_{2}. \end{array} \end{array}$$ (57)

Similarly, calculating the left eigenvector *V* = [*v*_1_, *v*_2_, *v*_3_, *v*_4_, *v*_5_, *v*_6_]^*T*^ with *V* × *J*(*E*_*q*0_) = 0, gives

$$\begin{array}{c} V_{i}\times J\left(E_{q0}\right)=0 \end{array}$$

Where

$$\begin{array}{c} V_{i}=(\begin{array}{cccccc} v_{1} & v_{2} & v_{3} & v_{4} & v_{5} & v_{6} \end{array}) \end{array}$$

$$\begin{array}{c} V_{i}\times \;(\begin{array}{cccccc} -\beta -\tau & 0 & -\frac{\phi \;\chi \;\Lambda }{\beta +\tau } & 0 & \zeta \;\lambda & 0\\ 0 & -\;\varsigma \;-\tau & \frac{\phi \;\chi \;\Lambda }{\beta +\tau } & 0 & 0 & 0\\ 0 & \Phi \;\;\varsigma \; & -\tau -\alpha -\psi & 0 & 0 & 0\\ 0 & 0 & -\omega \;\psi +\alpha +\psi & -\upsilon -\rho -\tau & 0 & 0\\ 0 & -(-1+\Phi )\;\varsigma \; & \omega \;\psi & \upsilon & -\tau -\lambda & 0\\ \beta & 0 & 0 & 0 & -(-1+\zeta )\lambda & -\tau \end{array}) \end{array}$$

$$\begin{array}{c} =(\begin{array}{c} 0\\ 0\\ 0\\ 0\\ 0\\ 0 \end{array}). \end{array}$$

Thus, We have

$$\{\begin{array}{ll} -(\beta +\tau )v_{1}+\beta \;v_{6} & =0\\ -(\;\varsigma \;+\tau )v_{2}+\Phi \;\;\varsigma \;\;v_{3}+(1-\Phi )\;\varsigma \;\;v_{5} & =0\\ -\frac{\phi \chi \Lambda }{\beta +\tau }v_{1}+\frac{\phi \chi \Lambda }{\beta +\tau }v_{2}-(\tau +\alpha +\Psi )v_{3}+(\omega \psi +\alpha +\psi )v_{4}+\omega \psi \;v_{5} & =0\\ -(\upsilon +\rho +\tau )v_{4}+\upsilon \;v_{5} & =0\\ \zeta \;\lambda \;v_{1}-(\tau +\lambda )v_{5}+(1-\zeta )\lambda \;v_{6} & =0\\ -\tau \;v_{6} & =0 \end{array}$$ (58)

Solving Eq (58), we get

$$\begin{array}{c} v_{1}=0 \end{array}$$ (59)

$$\begin{array}{c} v_{2}>0\;\;\;\;\left(can\;take\;any\;value\right) \end{array}$$ (60)

$$\begin{array}{c} v_{3}=\frac{\;\varsigma \;+\tau }{\Phi \;\;\varsigma \;}v_{2} \end{array}$$ (61)

$$\begin{array}{c} v_{4}=0 \end{array}$$ (62)

$$\begin{array}{c} v_{5}=0 \end{array}$$ (63)

$$\begin{array}{c} v_{6}=0 \end{array}$$ (64)

The stability of model (1) around SMADDFE is entirely determined by a and b defined as.

$$\begin{array}{c} a=\sum_{k,i,j=1}^{n=6}v_{k}\;w_{i}\;w_{j}\;\frac{\partial^{2}g_{x}\left(0,0\right)}{\partial y_{i}\;\partial y_{j}} \end{array}$$ (65)

$$\begin{array}{c} b=\sum_{k,i=1}^{n=6}v_{k}\;w_{i}\;\frac{\partial^{2}g_{x}\left(0,0\right)}{\partial y_{i}\;\partial \phi^{*}} \end{array}$$ (66)

Thus, the only non-zero terms in the sums for a and b are those that correspond to *k* = 2, 3. So from the system (47), we have

$$\begin{array}{c} g_{2}=\phi \;\chi \;y_{3}y_{1}-(\;\varsigma \;+\tau )y_{2} \end{array}$$ (67)

$$\begin{array}{c} g_{3}=\Phi \;\;\varsigma \;\;y_{2}-(\tau +\psi +\alpha )y_{3}. \end{array}$$ (68)

All the second derivatives of (67) are zeros. The only remaining Eq is (68) have non zero derivatives as

$$\begin{array}{c} \frac{\partial^{2}g_{2}}{\partial y_{1}\partial y_{3}}=\phi^{*}\;\chi \\ \frac{\partial^{2}g_{2}}{\partial y_{3}\partial y_{1}}=\phi^{*}\;\chi \\ \frac{\partial^{2}g_{2}}{\partial y_{3}\partial \phi }=y_{1}^{*}\;\chi \end{array}$$

Now by using (65), we have

$$\begin{array}{c} a=v_{2}\;w_{1}\;w_{3}\left(\frac{\partial^{2}g_{2}}{\partial y_{1}\partial y_{3}}+\frac{\partial^{2}g_{2}}{\partial y_{3}\partial y_{1}}\right) \end{array}$$

$$\begin{array}{c} a=2v_{2}\;w_{1}\;w_{3}\;\phi^{*}\;\chi <0 \end{array}$$ (69)

For the value of b, we use (66)

$$\begin{array}{c} b=v_{2}\;w_{3}\;x_{1}^{*}\;\chi >0 \end{array}$$ (70)

Since the unique endemic equilibrium point is locally asymptotically stable for **R**_0_ > 1 and model (1) displays forward bifurcation at **R**_0_ = 1, the SMADDFE and SMADEEP cannot coexist at **R**_0_ < 1.

## 7 Sensitivity analysis of SMAD model

We performed a sensitivity analysis to show the influence of every parameter on the SMAD transmission. We applied the normalized sensitivity index concept to conduct this analysis.

**Definition**

The normalized forward sensitivity index (NFSI) of a variable, threshold parameter **R**_0_, that depends differentially on a parameter A is defined as:

$$\begin{array}{c} \Lambda_{p}^{R_{0}}=\frac{\partial R_{0}}{\partial I_{1}}\times \frac{A}{R_{0}} \end{array}$$

Where s denotes all parameters and **R**_0_ is the basic reproduction number

$$\begin{array}{c} R_{0}=\frac{\Lambda \;\;\varsigma \;\;\phi \;\chi \;\Phi }{\left(\alpha +\psi +\tau )\;(\beta +\tau )\;(\;\varsigma \;+\tau \right)}. \end{array}$$

For calculating the sensitivity index of **R**_0_ to the parameters of the model:

- For the parameter *ς*
  $$\begin{array}{c} \Lambda_{\varsigma }^{R_{0}}=\frac{\partial R_{0}}{\partial \varsigma }\times \frac{\varsigma }{R_{0}}\\ \Lambda_{\varsigma }^{R_{0}}=\frac{\Lambda \;\Phi \;\chi \;\phi \;\tau }{\left(\beta +\tau ){\left(\tau +\varsigma \right)}^{2}(\tau +\alpha +\psi \right)}\times \frac{\left(\beta +\tau )(\tau +\varsigma )(\tau +\alpha +\psi \right)}{\Lambda \;\Phi \;\chi \;\phi }\\ \Lambda_{\varsigma }^{R_{0}}=\frac{\tau }{\tau +\varsigma }>0 \end{array}$$
- For the parameter *ϕ*
  $$\begin{array}{c} \Lambda_{\phi }^{R_{0}}=\frac{\partial R_{0}}{\partial \phi }\times \frac{\phi }{R_{0}}\\ \Lambda_{\phi }^{R_{0}}=\frac{\Lambda \;\;\varsigma \;\;\Phi \;\chi }{\left(\beta +\tau )(\tau +\;\varsigma \;)(\tau +\alpha +\psi \right)}\times \frac{\left(\beta +\tau )(\tau +\;\varsigma \;)(\tau +\alpha +\psi \right)}{\Lambda \;\;\varsigma \;\;\Phi \;\chi }\\ \Lambda_{\phi }^{R_{0}}=1>0 \end{array}$$
- For the parameter *χ*
  $$\begin{array}{c} \Lambda_{\chi }^{R_{0}}=\frac{\partial R_{0}}{\partial \chi }\times \frac{\chi }{R_{0}}\\ \Lambda_{\chi }^{R_{0}}=\frac{\Lambda \;\;\varsigma \;\;\Phi \;\phi }{\left(\beta +\tau )(\tau +\;\varsigma \;)(\tau +\alpha +\psi \right)}\times \frac{\left(\beta +\tau )(\tau +\;\varsigma \;)(\tau +\alpha +\psi \right)}{\Lambda \;\;\varsigma \;\;\Phi \;\phi }\\ \Lambda_{\chi }^{R_{0}}=1>0 \end{array}$$
- For the parameter Φ
  $$\begin{array}{c} \Lambda_{\Phi }^{R_{0}}=\frac{\partial R_{0}}{\partial \Phi }\times \frac{\Phi }{R_{0}}\\ \Lambda_{\Phi }^{R_{0}}=\frac{\Lambda \;\;\varsigma \;\;\chi \;\phi }{\left(\beta +\tau )(\tau +\;\varsigma \;)(\tau +\alpha +\psi \right)}\times \frac{\left(\beta +\tau )(\tau +\;\varsigma \;)(\tau +\alpha +\psi \right)}{\Lambda \;\;\varsigma \;\;\chi \;\phi }\\ \Lambda_{\Phi }^{R_{0}}=1>0 \end{array}$$
- For the parameter *β*
  $$\begin{array}{c} \Lambda_{\beta }^{R_{0}}=\frac{\partial R_{0}}{\partial \beta }\times \frac{\beta }{R_{0}}\\ \Lambda_{\beta }^{R_{0}}=-\frac{\Lambda \;\;\varsigma \;\;\Phi \;\chi \;\phi }{{\left(\beta +\tau \right)}^{2}(\tau +\;\varsigma \;)(\tau +\alpha +\psi )}\times \frac{\beta \;(\beta +\tau )(\tau +\;\varsigma \;)(\tau +\alpha +\psi )}{\Lambda \;\;\varsigma \;\;\Phi \;\chi \;\phi }\\ \Lambda_{\beta }^{R_{0}}=-\frac{\beta }{\beta +\tau }<0 \end{array}$$
- For the parameter *τ*
  $$\begin{array}{c} \Lambda_{\tau }^{R_{0}}=\frac{\partial R_{0}}{\partial \tau }\times \frac{\tau }{R_{0}}\\ \Lambda_{\tau }^{R_{0}}=-3\;\frac{\phi \;(\tau^{2}+2/3(\psi +\beta +\alpha +\;\varsigma \;)\tau +1/3(\psi +\beta +\alpha )\;\varsigma \;+1/3\;\beta \;(\psi +\alpha ))\chi \;\Phi \;\;\varsigma \;\;\Lambda }{{\left(\beta +\tau \right)}^{2}{\left(\tau +\;\varsigma \;\right)}^{2}{\left(\tau +\alpha +\psi \right)}^{2}} \end{array}$$
  $$\begin{array}{c} \times \frac{\tau \;(\beta +\tau )(\tau +\;\varsigma \;)(\tau +\alpha +\psi )}{\Lambda \;\;\varsigma \;\;\Phi \;\chi \;\phi } \end{array}$$
  $$\begin{array}{c} \Lambda_{\tau }^{R_{0}}=-3\;\frac{\tau \;(\tau^{2}+2/3(\psi +\beta +\alpha +\;\varsigma \;)\tau +1/3(\psi +\beta +\alpha )\;\varsigma \;+1/3\;\beta \;(\psi +\alpha ))}{\left(\tau +\alpha +\psi )(\beta +\tau )(\tau +\;\varsigma \;\right)}<0 \end{array}$$
- For the parameter *α*
  $$\begin{array}{c} \Lambda_{\alpha }^{R_{0}}=\frac{\partial R_{0}}{\partial \alpha }\times \frac{\alpha }{R_{0}}\\ \Lambda_{\alpha }^{R_{0}}=-\frac{\Lambda \;\;\varsigma \;\;\Phi \;\chi \;\phi }{(\beta +\tau )(\tau +\;\varsigma \;){\left(\tau +\alpha +\psi \right)}^{2}}\times \frac{\alpha \;(\beta +\tau )(\tau +\;\varsigma \;)(\tau +\alpha +\psi )}{\Lambda \;\;\varsigma \;\;\Phi \;\chi \;\phi }\\ \Lambda_{\alpha }^{R_{0}}=-\frac{\alpha }{\tau +\alpha +\psi }<0 \end{array}$$
- For the parameter *ψ*
  $$\begin{array}{c} \Lambda_{\psi }^{R_{0}}=\frac{\partial R_{0}}{\partial \psi }\times \frac{\psi }{R_{0}}\\ \Lambda_{\psi }^{R_{0}}=-\frac{\Lambda \;\;\varsigma \;\;\Phi \;\chi \;\phi }{(\beta +\tau )(\tau +\;\varsigma \;){\left(\tau +\alpha +\psi \right)}^{2}}\times \frac{\psi \;(\beta +\tau )(\tau +\;\varsigma \;)(\tau +\alpha +\psi )}{\Lambda \;\;\varsigma \;\;\Phi \;\chi \;\phi }\\ \Lambda_{\psi }^{R_{0}}=-\frac{\psi }{\tau +\alpha +\psi }<0 \end{array}$$

**Fig 2:**
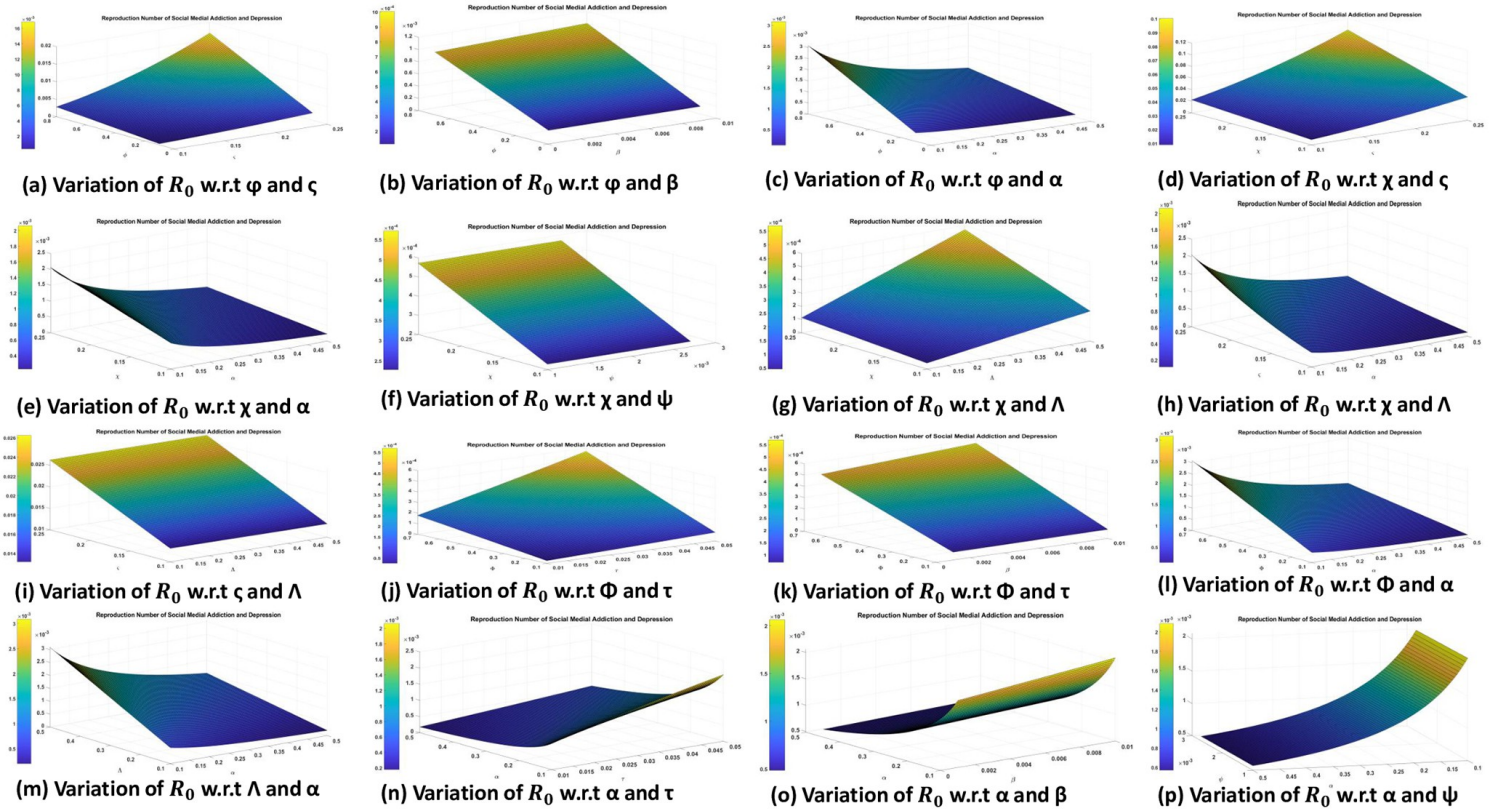
Variation of different parameters on the R_0_.

The sensitivity index of the **R**_0_ w.r.t key parameters is given in Table 2. If any of the variables have positive indices, it means that they are having a significant influence on the spread of the disease throughout the population. Additionally, when parameter values increase, individuals whose sensitivity indices are negative have an impact on reducing disease burden in the population. As a result, stakeholders and policy makers should concentrate on decreasing positive indices and raising negative indices parameters. The impact of different parameters is shown in Fig 2. The graph in Fig 2(a) shows how the value of the **R**_0_ changes when the number of people who leave the exposed class *ς* and the pace at which addiction spreads to the vulnerable population *ϕ* fluctuate. Here, we see that *ς* raises the value of **R**_0_ as *ϕ* increases, indicating that the disease can be eradicated by lowering *ς* and *ϕ*. Fig 2(a) also shows that *ϕ* has a stronger effect on **R**_0_ than *ς*. In Fig 2(b), the plot depicts how the value of **R**_0_ changes when the susceptible who avoid and/or stop using social media *β* and the transmission rate of addiction to the susceptible population *ϕ* varies concurrently. Here we analyses that *ϕ* increases the value of **R**_0_ and *β* decreases the value of **R**_0_, which indicates that the disease can be extinct by reducing the value of *ϕ* and by increasing the value of *β*. From Fig 2(b), it can also be observed that *ϕ* has more influence than beta on **R**_0_. Also, the graph in Fig 2(e) shows how the value of the **R**_0_ evolves when the contact rate of susceptible with the addicted population *χ* and the rate at which depression is caused by media effect *α* vary simultaneously. Here, we determine that *χ* increases the **R**_0_ value while alpha reduces it, indicating that the disease may be wiped out by decreasing *χ* and raising *α*. It is also clear from Fig 2(e) that *χ* has a stronger impact on **R**_0_ than *α*. Similarly, the graph in Fig 2(j) shows how the value of **R**_0_ varies when the natural mortality rate *τ* and the proportion of individuals exposed who subsequently develop addiction Φ are taken into account. Here, we see that *τ* reduces the value of **R**_0_ but Φ increases it, indicating that the disease may be cured by decreasing Φ. Fig 2(j) additionally indicates that Φ has a stronger impact on **R**_0_ than *τ*.

**Table 2:** Sensitivity index of R_0_.

Parameters	Sensitivity index	Values
*ς*	+ev	0.16666
*ϕ*	+ev	1
*χ*	+ev	1
Φ	+ev	1
*β*	-ev	-0.0105
*τ*	-ev	-1.04
*α*	-ev	-0.4
*ψ*	-ev	-0.56

### 7.1 Global sensitivity analysis by partial rank correlation coefficient PRCC

Sensitivity analysis is used to identify key parameters that influence the model’s predictions and could potentially reduce the spread of the disease. To accomplish this, the Latin Hypercube Sampling (LHS) method is used here, to estimate the PRCC, which is a global sensitive analysis method [51]. The level of correlation between the model output and input variables is evaluated using PRCC. It measures the linear dependency between two variables after taking into account the linear impacts of other variables.

The PRCC analysis is used here to examine the sensitivity of the model parameters on the output variables of social media addiction and depression. It measures how much the variation in the output variables is explained by the variation in the input parameters while controlling for the effects of other input parameters. The range of PRCC values is [-1 1], where a value of 1 specifies a perfect positive correlation, -1 specifies a perfect negative correlation, and 0 indicates no correlation between the variables.

**Fig 3:**
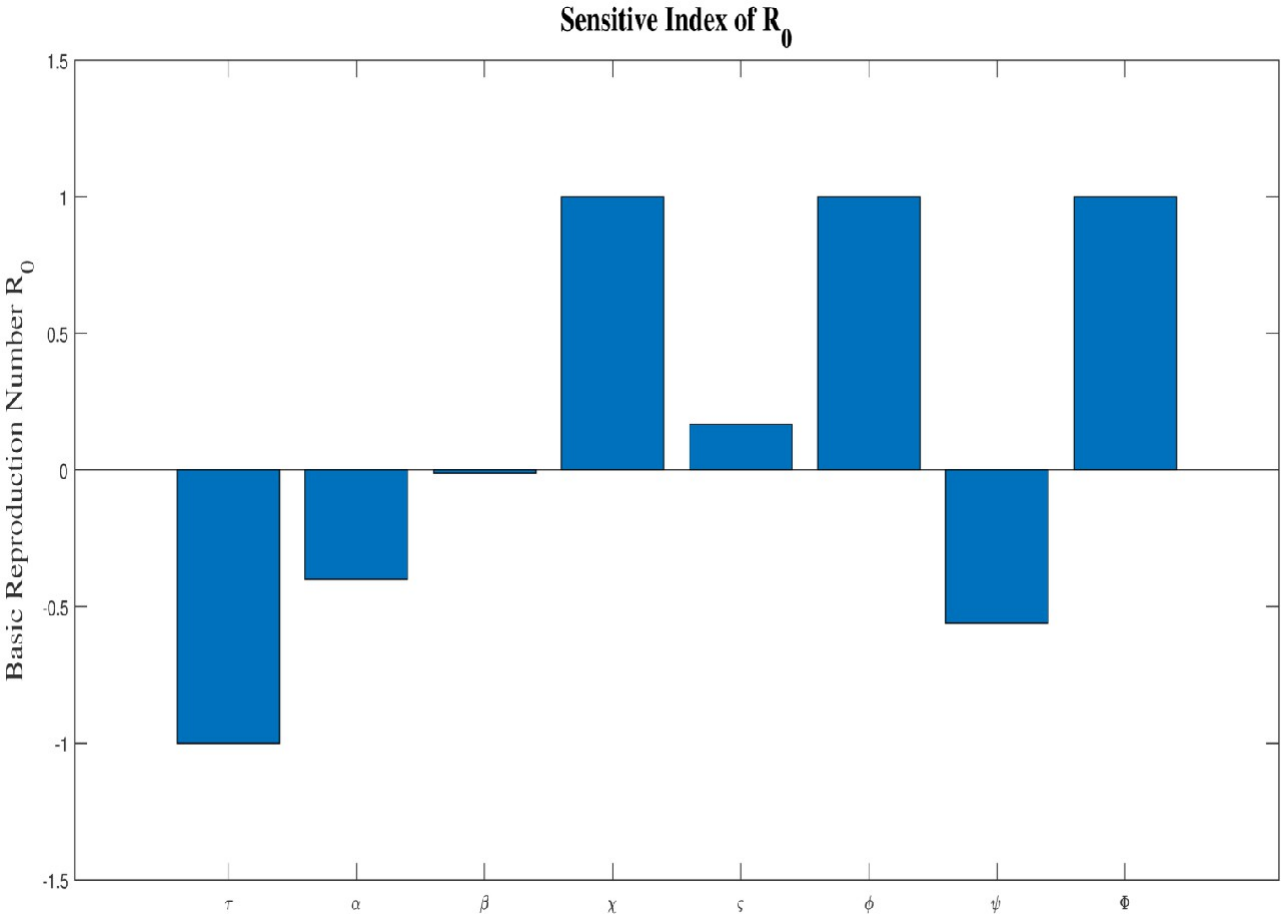
Sensitivity Index Of Reproduction Number R_0_.

**Fig 4:**
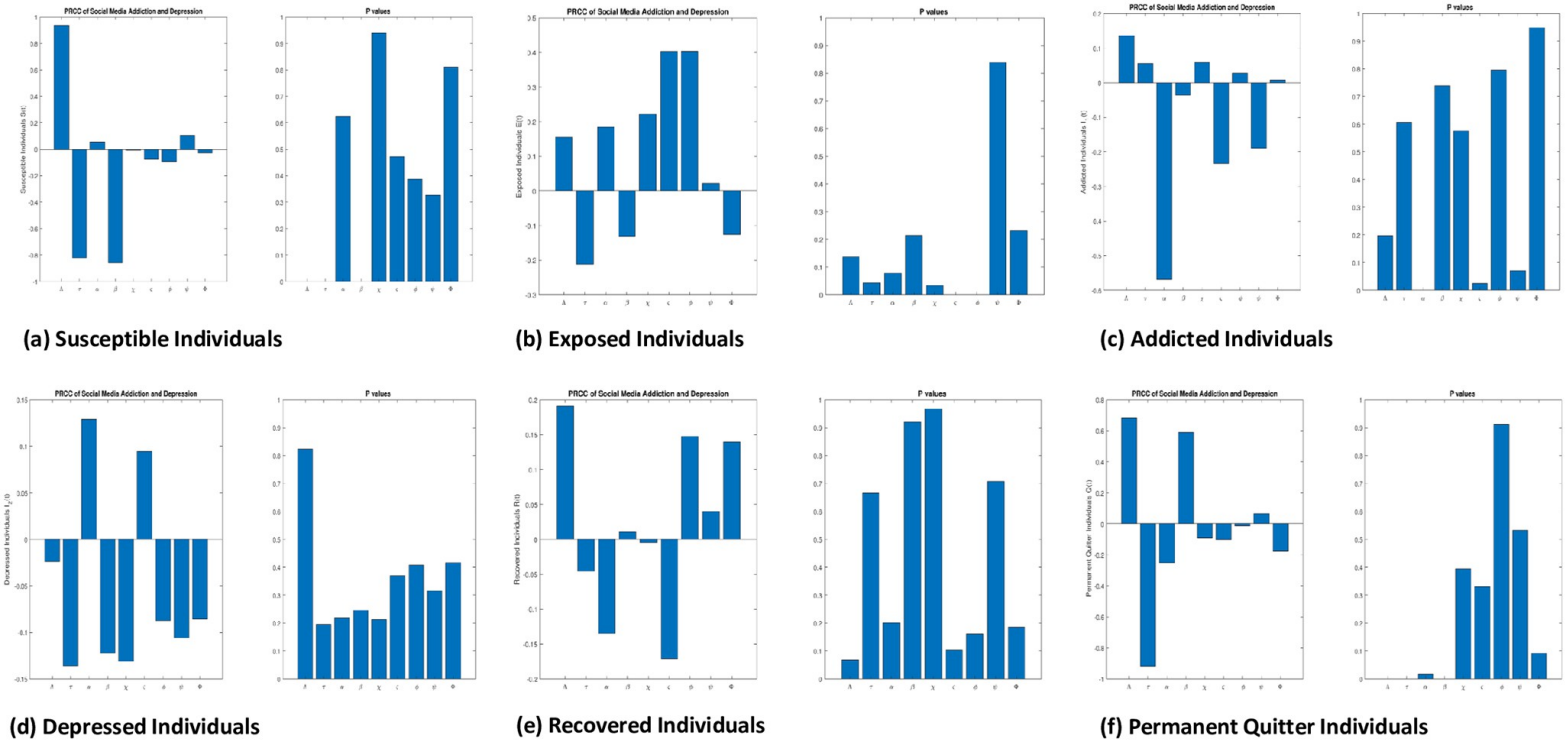
PRCC values representing the sensitivities of the model outputs with respect to R_0_.

For each output variable of interest, approximately 5,000 simulations were conducted, with each model input parameter allocated a uniform distribution from its baseline value. Now, we take into consideration the model outputs are the number of susceptible individuals, exposed individuals, addicted individuals, depressed individuals, recovered Individuals,’ permanent quitter individuals’, and **R**_0_.Fig 3 indicates the sensitivity indexes of parameters of **R**_0_. Fig 4 refers to the correlation between the parameters of **R**_0_ and all model classes. It also indicates the P-values.

By performing PRCC analysis, we aimed to identify the most influential parameters in the SMAD model and to determine the direction and strength of their influence on the output variables. From Fig 4c and 4d, we analyse that for addicted individuals the parameters Λ, *τ*, *ϕ*, *ψ*, Φ have positive PRCC values and *α*, *β*, *χ*, *ς* have negative PRCC values. Similarly for depressed individuals, the parameters *α*, *ϕ*, *ψ* have positive PRCC values, and Λ, *τ*, Φ, *β*, *χ*, *ς* have negative PRCC values. This analysis indicates that a positive change in those parameters having positive PRCC values will increase the number of infected and depressed individuals. On the other hand, those parameters have negative PRCC values, which shows that increasing these parameters will decrease the number of infected and depressed individuals.

## 8 Numerical results of SMAD model

This section carries out a detailed numerical interpretation of the model (1) by RK-4 scheme using Matlab programming language. We now analyse numerical experiments for this technique using all the parameter values listed in the Table 1. To verify the accuracy of all the solutions of the model (1), the Rk-4 scheme applied using the discretization step size *h* = 0.001 for DFE *E*_*q*0_ and EEP *E*_*q*1_. It is observe that numerical scheme converge to true steady states(of *E*_*q*0_ and *E*_*q*1_) for 1 < **R**_0_ < 1 and give all positive solutions in feasible region Δ with IC’s (*S*(0), *E*(0), *I*_1_(0), *I*_2_(0), *R*(0), *Q*(0)) = (100, 1, 5, 2, 0, 10).

### 8.1 Case 1: Numerical simulation for R_0_ < 1

**Fig 5:**
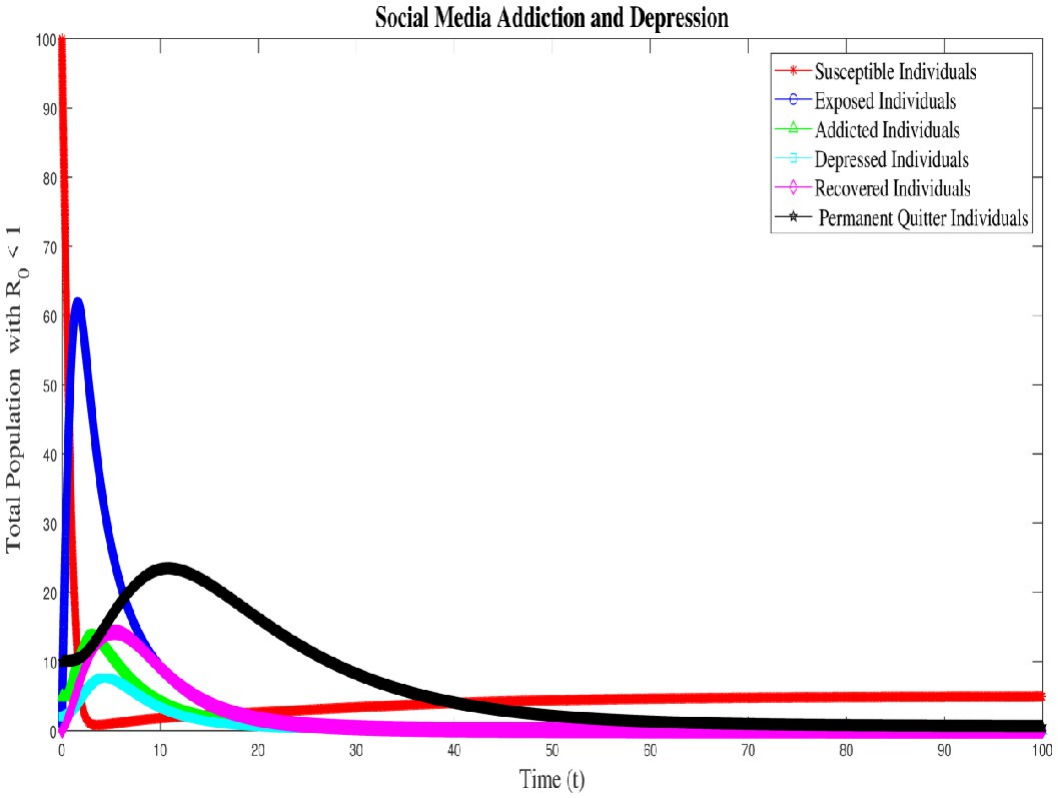
Solution of social media addiction and depression model (SMAD) obtained by RK-4 scheme for *E*_*q*0_ with IC’s and step size h = 0.001. Numerical scheme converge to true equilibrium point *E*_*q*0_ with **R**_0_ < 1.

**Fig 6:**
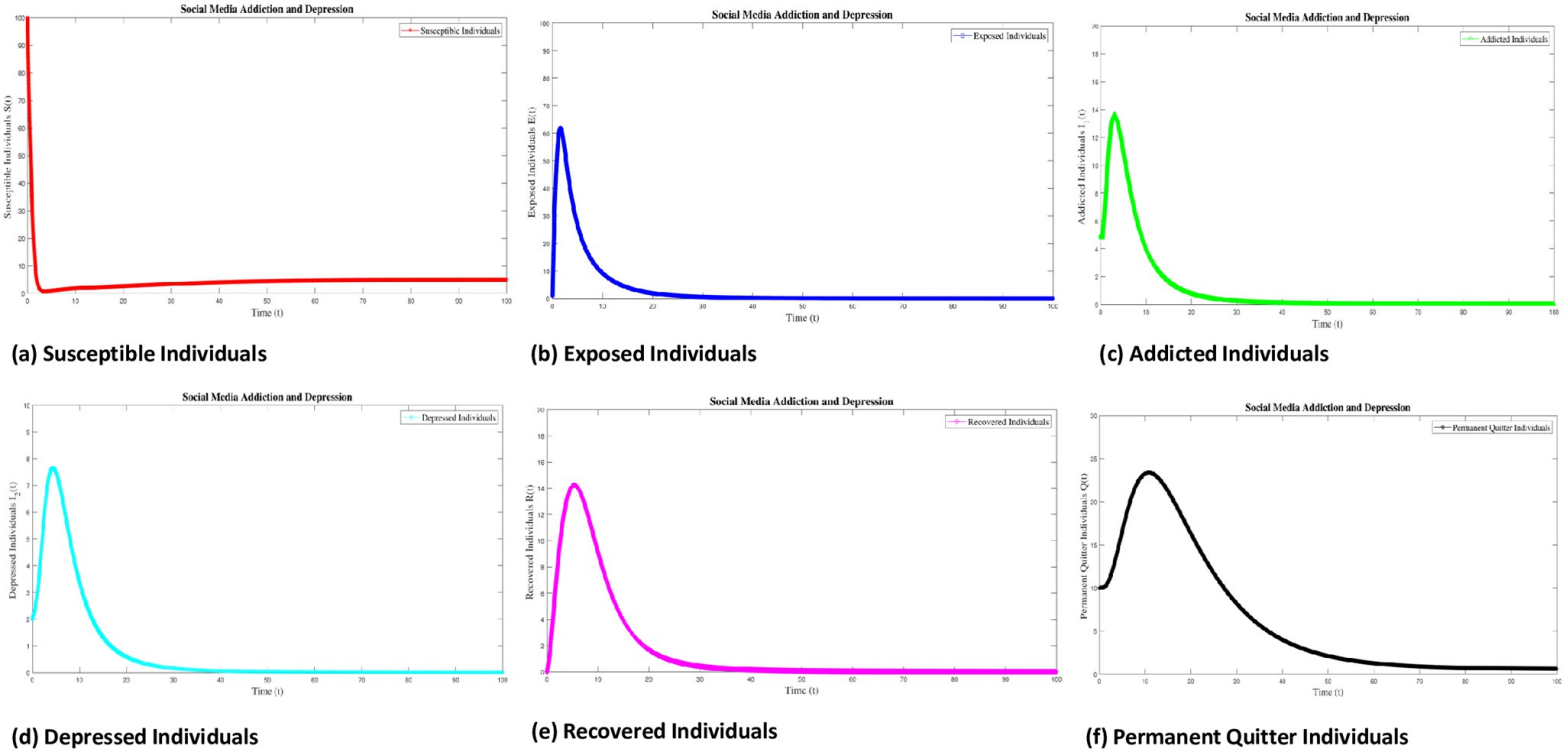
Seperate plots of all classes of solution of social media addiction and depression model (SMAD) obtained by RK-4 scheme for *E*_*q*0_ with IC’s and step size h = 0.001. Numerical scheme converge to true equilibrium point *E*_*q*0_ with **R**_0_ < 1.

**Fig 7:**
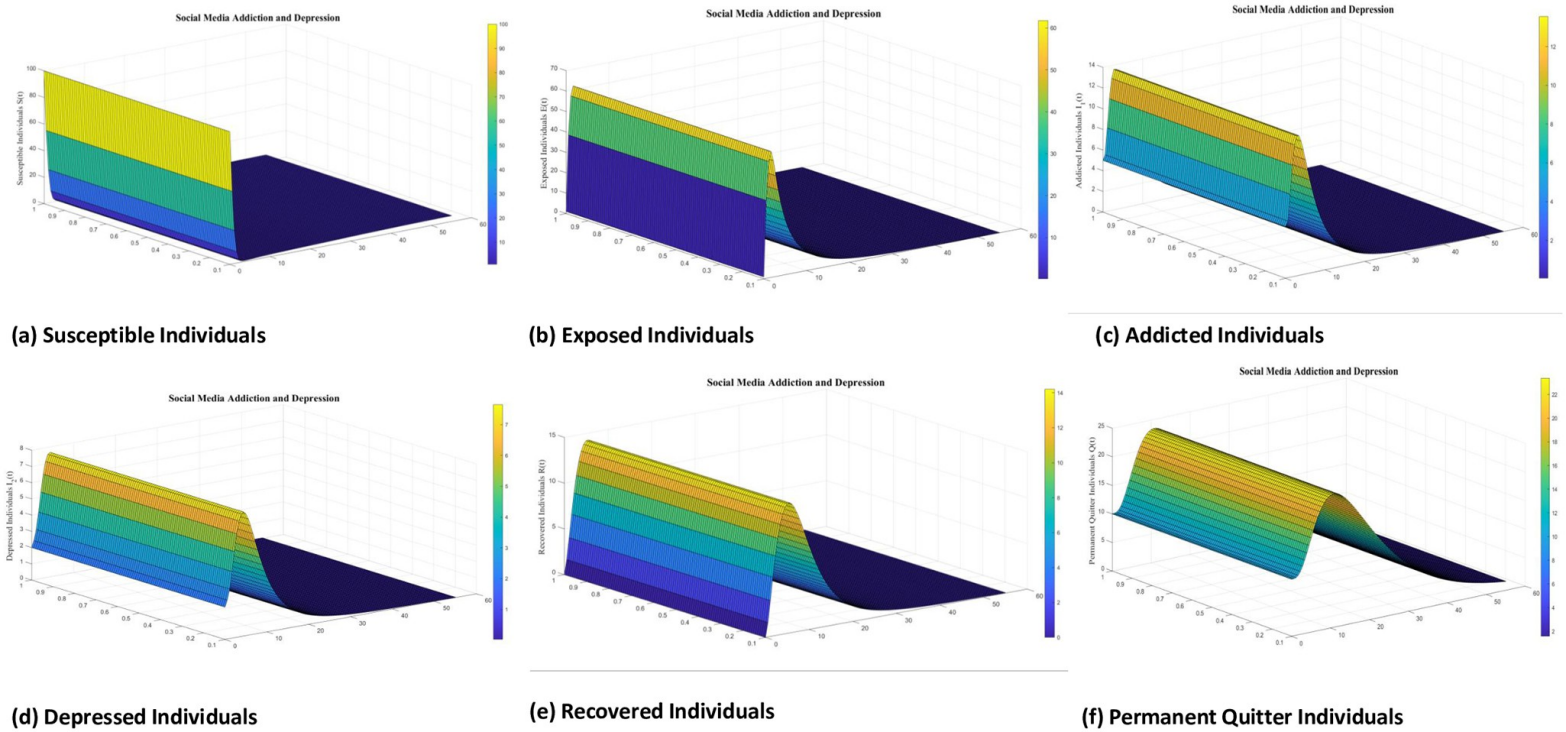
Separate surface plots of solution of all classes of social media addiction and depression model (SMAD) obtained by RK-4 scheme for *E*_*q*0_ with IC’s and step size h = 0.001. Numerical scheme converge to true equilibrium point *E*_*q*0_ with **R**_0_ < 1.

Numerical interpenetration for **R**_0_ < 1 is discussed here. Based on the numerical results of the SAMD model with RK-4 scheme with N = 1000 and IC’s, if we take Λ = 0.5, *χ* = 0.25, *ϕ* = 0.8, *ς* = 0.25, Φ = 0.7, *β* = 0.01, *τ* = 0.09; *α* = 0.5, *ψ* = 0.0027 then **R**_0_ = 0.8684 < 1 obtained and the spread of disease (addiction and depression) stop which can be shown in Figs 5 and 6. In this case the DFE is *E*_*q*0_=(5 0 0 0 0 0.5556). As time passes, we see that the populations of exposed and addicted people quickly expand and then gradually decline to zero. Depression transmission also increases along with the number of addicts. As more people recover from addiction and depression, there are more recovering populations. At the termination of the addicts transmission, the number of depressed people also quits, and the population of those who have recovered from addiction falls to zero. Additionally, the number of permanent quitters fluctuates over time. This is due to the variations in the number of susceptible and recovered populations. As the number of depressed individuals decreases with the decrease of addicted individuals, the permanent quitter increases until a stable equilibrium is reached at 0.5556. This variation is due to some individuals rejoining the exposed class after recovery and those individuals who leave the treatment of depression or need a second dose moved toward the exposed and/or addicted class. On the other side, infected people (addicted and depressed) decreased, which along with the decrease in exposed and permanent quitters once again increased the number of high-quality people. This could be the result of infected persons (addicted and depressed) getting well or new people joining the susceptible population who are not addicted or depressed and are balanced and stable at 5. The surface plot of the SMAD model is shown in Fig 7.

### 8.2 Case 2: Numerical simulation for R_0_ > 1

**Fig 8:**
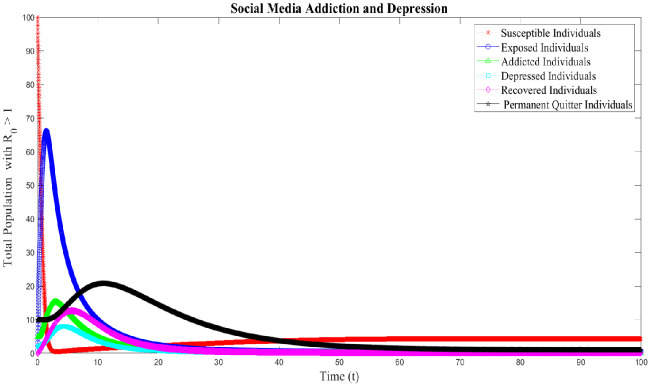
Solution of social media addiction and depression model (SMAD) obtained by RK-4 scheme for *E*_*q*1_ with IC’s and step size h = 0.001. Numerical scheme converge to true equilibrium point *E*_*q*1_ with **R**_0_ > 1.

**Fig 9:**
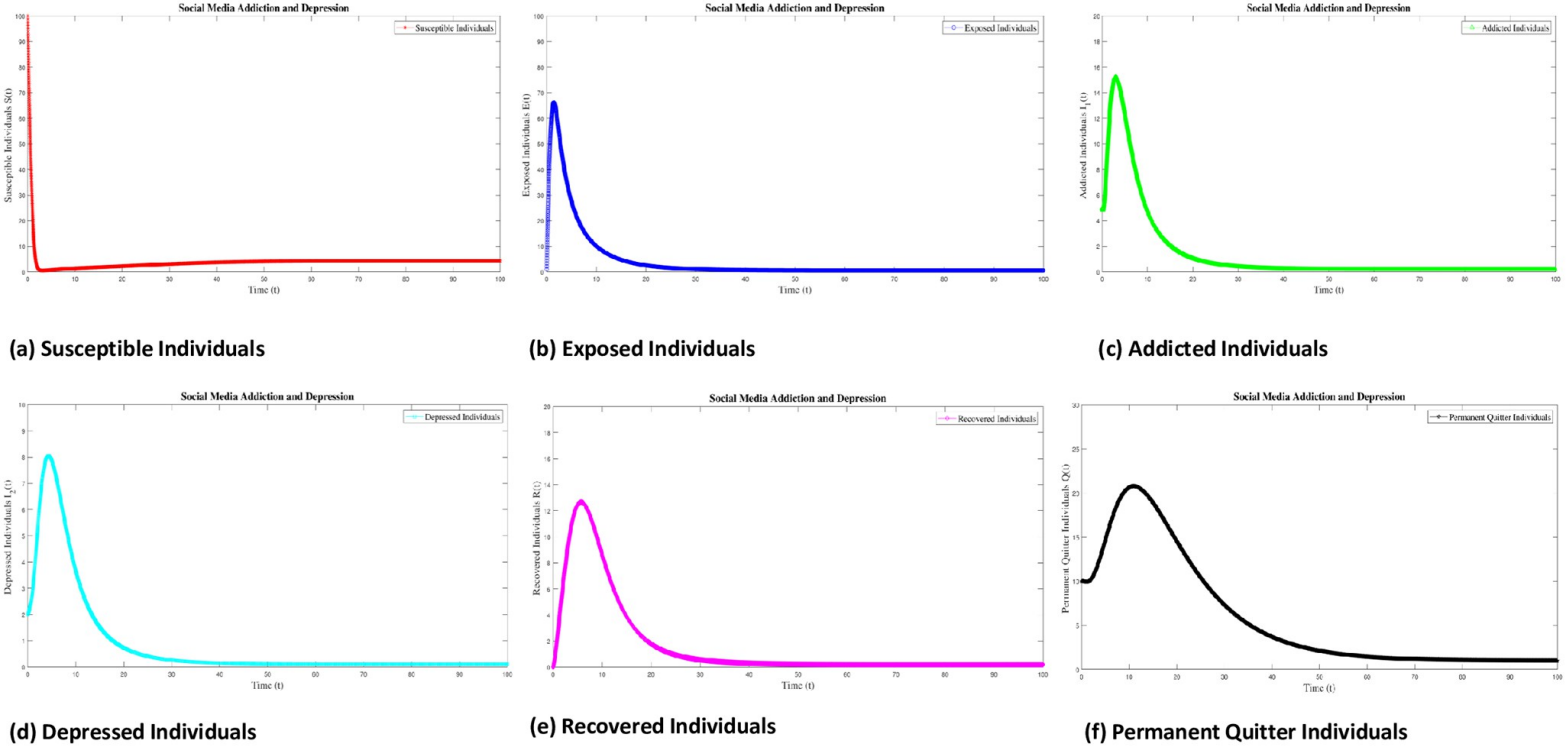
Separate plots of solution of all classes of social of social media addiction and depression model (SMAD) obtained by RK-4 scheme for *E*_*q*1_ with IC’s and step size h = 0.001. Numerical scheme converge to true equilibrium point *E*_*q*1_ with **R**_0_ > 1.

**Fig 10:**
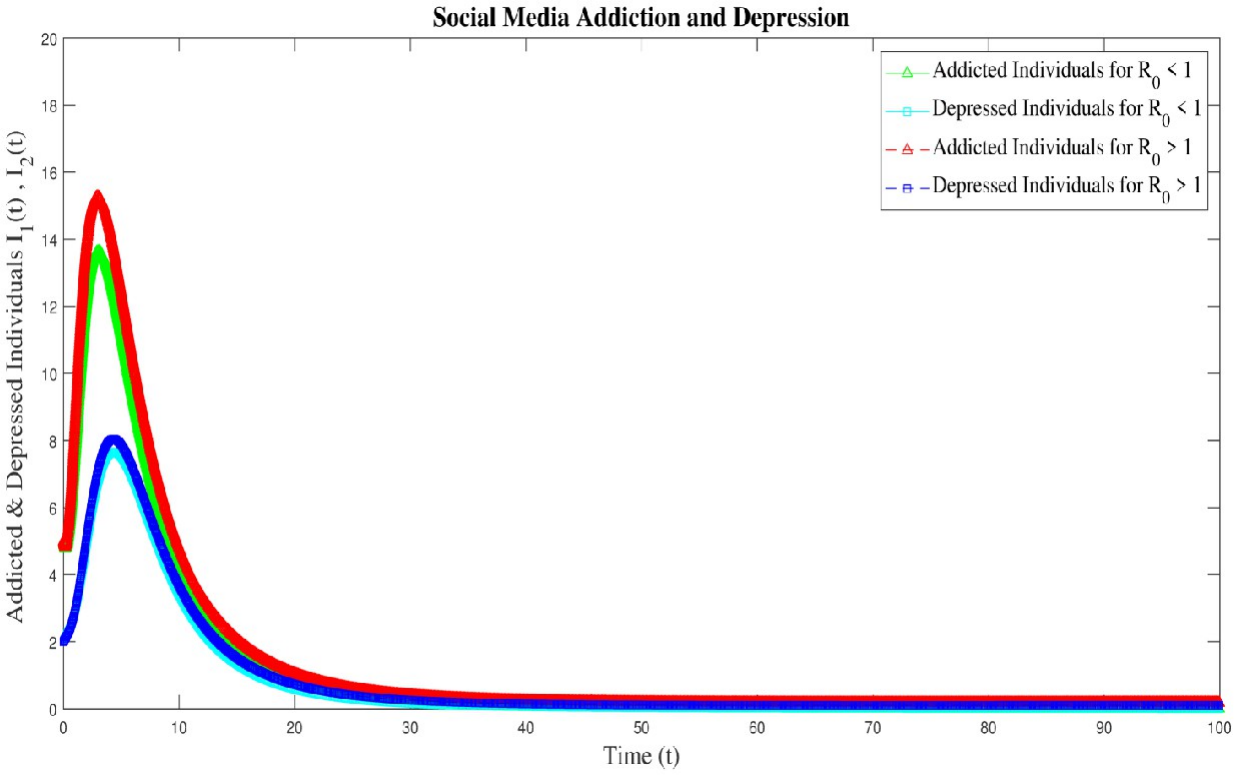
Comparison of addicted and depresses individuals of social media addiction and depression (SMAD) model at R_0_ < 1 and R_0_ > 1.

**Fig 11:**
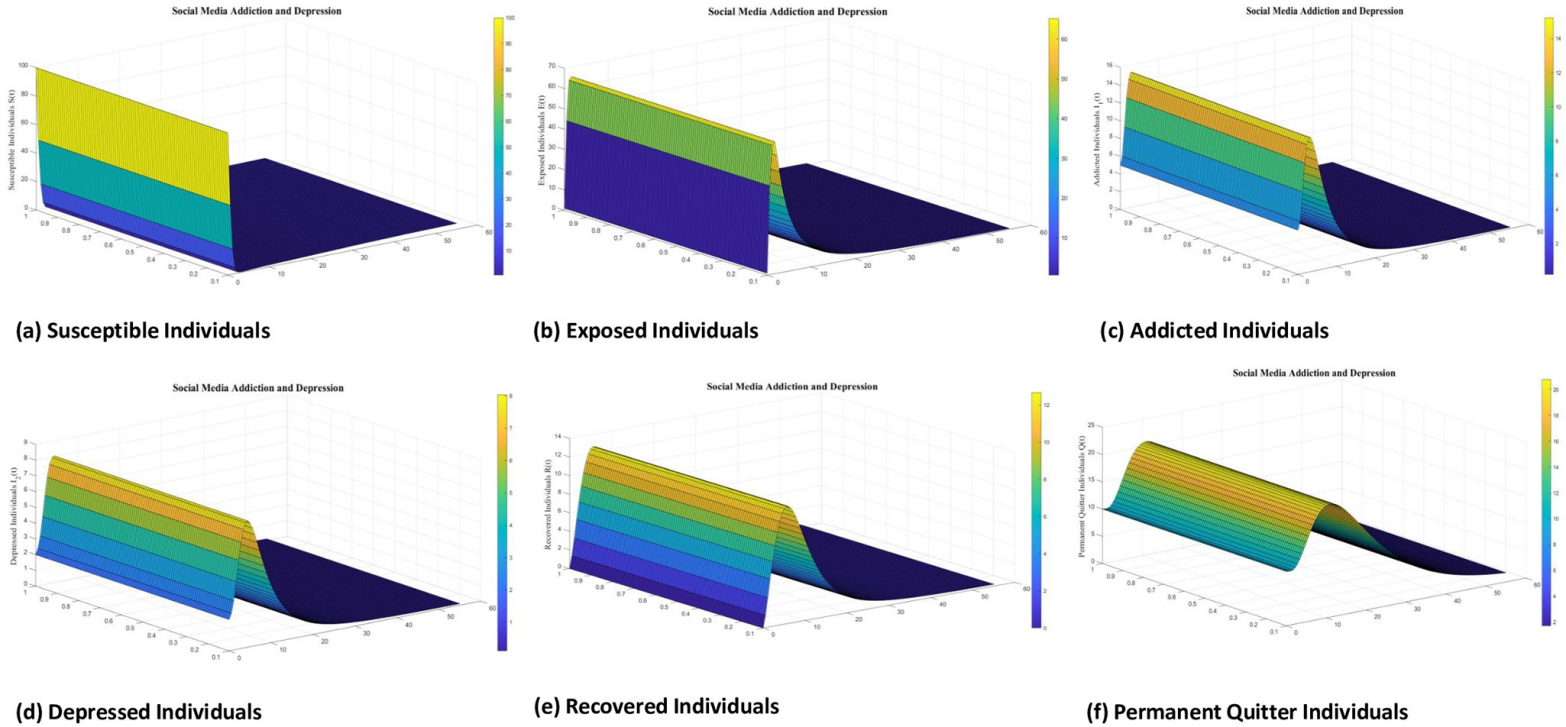
Separate surface plots of solution of all classes of social of social media addiction and depression model (SMAD) obtained by RK-4 scheme for *E*_*q*1_ with IC’s and step size h = 0.001. Numerical scheme converge to true equilibrium point *E*_*q*1_ with **R**_0_ > 1.

In this section, we discussed the numerical interpenetration for **R**_0_ > 1. The numerical results of the SAMD model with RK-4 scheme with N = 1000 and ICs are shown in Figs 8 and 9. If we take Λ = 0.60556, *χ* = 0.26104, *ϕ* = 0.89339, *ς* = 0.22874, *φ* = 0.80142, *β* = 0.010014, *τ* = 0.095, *α* = 0.47155, *ψ* = 0.002909 then **R**_0_ = 1.3372 > 1 obtained and the EEP is *E*_*q*1_=(4.3123 0.5588 0.1799 0.1055 0.2013 1.0055). These Figs 8 and 10 indicates that if **R**_0_ > 1, there is a sharp initial increase in exposed and addicted individuals. Once the addiction reaches its peak, the number of exposed and addicted individuals stabilizes at 0.5588 and 0.1799, respectively. Due to the rapid increase in addiction, the depressed individual also increased and gradually decreased and stable at 0.1055. Also, as the decrease of exposed individuals along with addicted and depressed, the recovered individuals fluctuated over time and stable at 0.2013. With the treatment of addicted and depressed people, permanent quitters also increase and gradually decrease tending to a stable point of 1.0055. Conversely, as the exposed and permanent quitter increases, the susceptible individual decreases and then stable with a slight increase at 4.3123. The surface plot of the SMAD model is shown in Fig 11. Also, the comparison of addicted and depressed individuals for **R**_0_ > 1 and **R**_0_ < 1 are presented in Fig 10.

### 8.3 Variation of parameters

The focus of this section is to discuss the effects of different parameters on the SMAD model. In the numerical experiments, we are varying a few parameters, while keeping all other parameters fixed. Here we represent the variation of the Percentage of those who are exposed who later become addicts Φ, the transmission rate of addiction to the susceptible individuals *ϕ*, the rate at which depression is brought on by media impact *α* and natural death rate *τ*.

#### 8.3.1 Variation of parameter Φ

**Fig 12:**
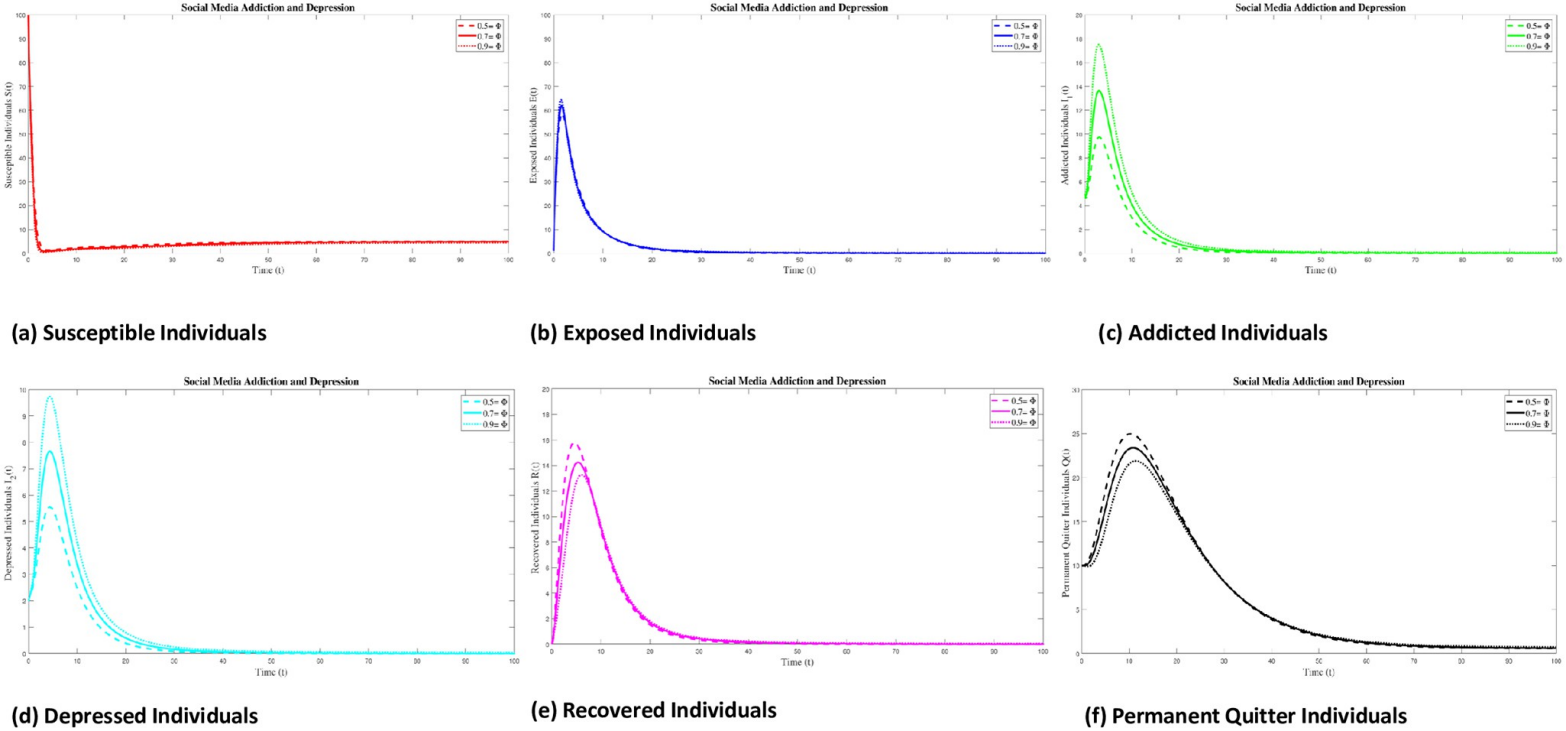
Numerical results of social media addiction and depression (SMAD) at different values of Φ = (0.5, 0.7, 0.9).

Here we represent the variation of the Percentage of those who are exposed and later become addicts Φ by remaining fixed other parameter values. We approximate the numerical result of the SMAD model at Φ = (0.5, 0.7, 0.9). From Fig 12c and 12d, we see that by increasing the value of Φ, exposed individual decreases but addicted and depressed individual increases respectively.

#### 8.3.2 Variation of parameter *τ*

**Fig 13:**
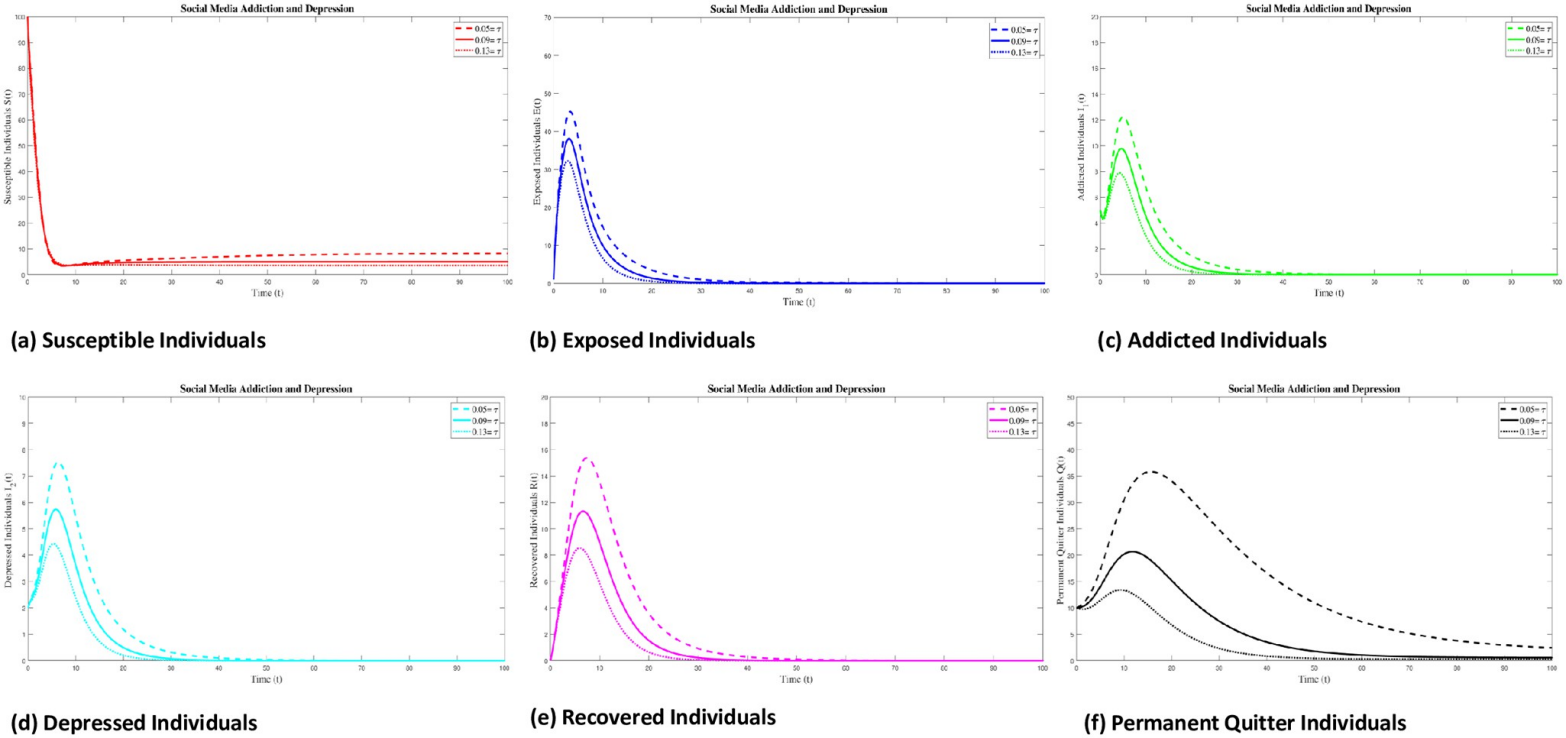
Numerical results of socail media addiction and depression (SMAD) at different values of *τ* = (0.05, 0.09, 0.13).

Here we show the variation of natural death rate *τ* by remaining fixed other parameter values. We approximate the numerical result of the SMAD model at *τ* = (0.05, 0.09, 0.13). From Fig 13, we see that by decreasing the value of *τ*, the individual of all classes increases.

#### 8.3.3 Variation of parameter *ϕ*

**Fig 14:**
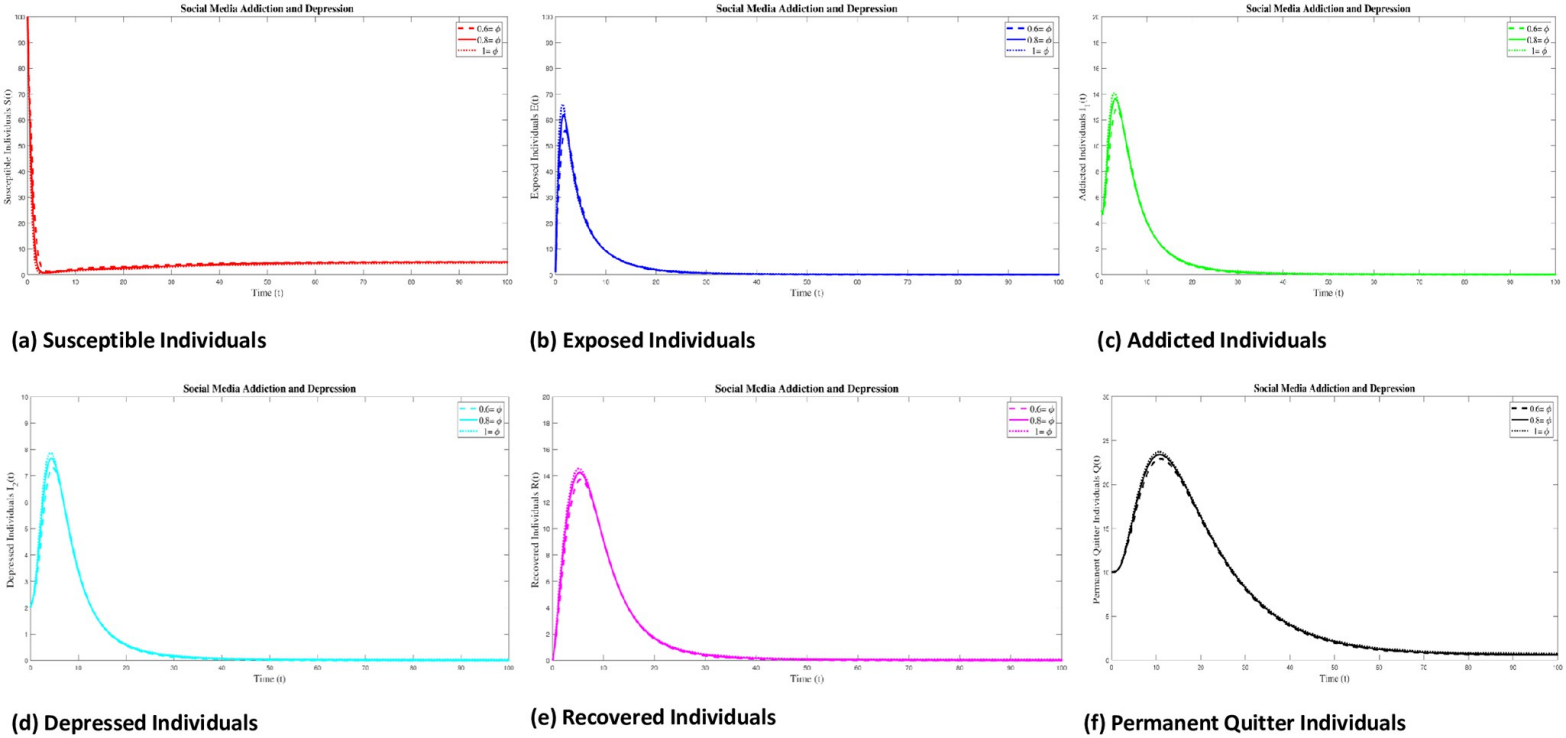
Numerical results of social media addiction and depression (SMAD) at different values of *ϕ* = (0.6, 0.8, 1).

Here we perform the variation of the transmission rate of addiction to the susceptible individuals *ϕ* by remaining fixed other parameter values. We approximate the numerical result of the SMAD model at *ϕ* = (0.6, 0.8, 1). From Fig 14c and 14d, we see that by increasing the value of *ϕ*, susceptible individual decrease but addicted and depressed individual increases.

#### 8.3.4 Variation of parameter *α*

**Fig 15:**
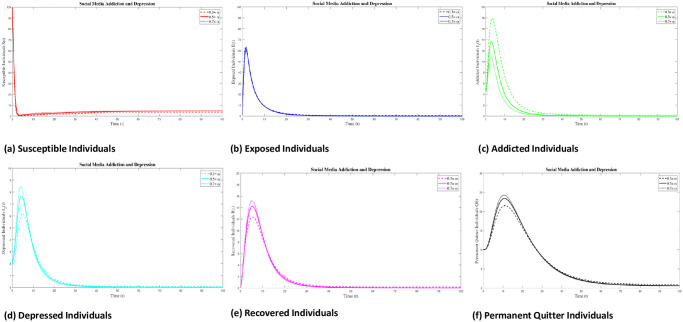
Numerical results of social media addiction and depression (SMAD) at different values of *α* = (0.3, 0.5, 0.7).

Here we describe the variation of the rate at which media influence causes people to become depressed *α* by remaining fixed on other parameter values. We approximate the numerical result of the SMAD model at *α* = (0.3, 0.5, 0.7). From Fig 15c and 15d, we see that by increasing the value of *α*, addictive individual decrease but depressed individual increases.

## 9 Conclusion

The primary objective of the article is to examine the depressive tendencies brought on by addiction to social media. A substantial body of literature has been utilized to examine social media issues and describe the impacts of social media addiction. The consequences of depression are not included in any of these models, which instead focus on the addition and control strategies of social media. We developed a social media addiction with depression model based on the prior hypotheses. We believed the population’s interplay with addiction and sadness to be more realistic. The threshold parameter **R**_0_ and equilibrium points (DFE and EEP) have been calculated. The local and global stability of DFE and EEP has been examined. The analysis indicates that if **R**_0_ < 1 then DFE is locally stable and if **R**_0_ > 1 then EEP is locally stable. The bifurcation study shows that the model displays forward bifurcation at **R**_0_ = 1. The uniqueness and existence of the proposed SMAD model were examined using the Lipschitz condition. To check the impact of different parameters, sensitivity analysis was performed by applying the definition of NFSI and PRCC techniques. Fourth Order Runge-Kutta technique estimates the approximate solutions for the proposed SMAD model. The results of a numerical simulation for the spread of addiction and depression in the cases of **R**_0_ < 1 and **R**_0_ > 1 show that the system is stable in both cases at its fixed points. With the increase in exposed individuals, the transmission of addiction increases due to which depressed individuals increase. A comparison of results of both cases indicates that infection (addiction and depression) increased when **R**_0_ > 1. Variation of specific parameters shows the influence on SMAD model performance. From an epidemiological perspective, this study demonstrates that any time the related reproduction number is reduced to (and kept at) a value smaller than unity, the disease under consideration may be eradicated from the community. Every time the rate of reproduction reaches one, the disease will continue to spread across the population. Environmental elements including seasonality, wealth factor, interaction rate, age structure, and optimal control can be added to this study in the future.
